# Comparative RNA‐Seq transcriptome analyses reveal distinct metabolic pathways in diabetic nerve and kidney disease

**DOI:** 10.1111/jcmm.13136

**Published:** 2017-03-08

**Authors:** Lucy M. Hinder, Meeyoung Park, Amy E. Rumora, Junguk Hur, Felix Eichinger, Subramaniam Pennathur, Matthias Kretzler, Frank C. Brosius, Eva L. Feldman

**Affiliations:** ^1^ Department of Neurology University of Michigan Ann Arbor MI USA; ^2^ Department of Internal Medicine University of Michigan Ann Arbor MI USA; ^3^ Department of Computational Medicine and Bioinformatics University of Michigan Ann Arbor MI USA; ^4^ Department of Molecular and Integrative Physiology University of Michigan Ann Arbor MI USA; ^5^ Department of Biomedical Sciences School of Medicine and Health Sciences University of North Dakota Grand Forks ND USA

**Keywords:** type 2 diabetes, diabetic peripheral neuropathy, diabetic nephropathy, pioglitazone

## Abstract

Treating insulin resistance with pioglitazone normalizes renal function and improves small nerve fibre function and architecture; however, it does not affect large myelinated nerve fibre function in mouse models of type 2 diabetes (T2DM), indicating that pioglitazone affects the body in a tissue‐specific manner. To identify distinct molecular pathways regulating diabetic peripheral neuropathy (DPN) and nephropathy (DN), as well those affected by pioglitazone, we assessed DPN and DN gene transcript expression in control and diabetic mice with or without pioglitazone treatment. Differential expression analysis and self‐organizing maps were then used in parallel to analyse transcriptome data. Differential expression analysis showed that gene expression promoting cell death and the inflammatory response was reversed in the kidney glomeruli but unchanged or exacerbated in sciatic nerve by pioglitazone. Self‐organizing map analysis revealed that mitochondrial dysfunction was normalized in kidney and nerve by treatment; however, conserved pathways were opposite in their directionality of regulation. Collectively, our data suggest inflammation may drive large fibre dysfunction, while mitochondrial dysfunction may drive small fibre dysfunction in T2DM. Moreover, targeting both of these pathways is likely to improve DN. This study supports growing evidence that systemic metabolic changes in T2DM are associated with distinct tissue‐specific metabolic reprogramming in kidney and nerve and that these changes play a critical role in DN and small fibre DPN pathogenesis. These data also highlight the potential dangers of a ‘one size fits all’ approach to T2DM therapeutics, as the same drug may simultaneously alleviate one complication while exacerbating another.

## Introduction

Type 2 diabetes mellitus (T2DM) affects over 387 million people worldwide 1, and its prevalence continues to increase 2. T2DM itself is a complex metabolic disease characterized by hyperglycaemia, hyperlipidemia and impaired insulin signalling that develops as a result of genetic factors, obesity or the environment. As T2DM progresses, oxidative stress, high circulating blood glucose levels and hyperlipidemia can promote microvascular complications that can result in severe debility and increased mortality. These complications are one of the greatest challenges facing the healthcare industry: in 2014 alone, the global medical expenditure for diabetic patients totalled over $245 billion, with 25–45% of those costs related to associated vascular complications 3.

The most common of these microvascular complications includes diabetic peripheral neuropathy (DPN) and diabetic nephropathy (DN) 4, 5. DPN affects 50% of diabetic patients and is characterized by progressive loss of sensation in the limbs, pain and allodynia. DPN progression also increases the risk of infection and foot ulcers that can lead to amputation of the affected limb 6. There is no cure for DPN, and treatments are limited to glycemic control and symptomatic relief 4. Similarly, DN affects approximately 40% of diabetic patients. Marked by albuminuria and impaired glomerular filtration, DN is the leading cause of end‐stage renal disease in the United states 7 and is primarily responsible for the increased mortality in T2DM 8. Thus, there is a critical need for effective therapeutics and a better understanding of the mechanisms underlying T2DM complications.

Pioglitazone is a drug that is often prescribed to treat T2DM 9, 10. In T2DM animal models, pioglitazone ameliorates DN and diabetic retinopathy *via* multiple pathways 9, 11, 12, 13, 14, 15, 16 and can attenuate neuropathic pain and nervous system inflammation 17, 18. Mechanistically, pioglitazone acts as an agonist of peroxisome proliferator‐activated receptor gamma (PPARG), but it differentially regulates metabolism in a tissue‐specific manner 19. We recently reported that pioglitazone normalized the renal function and significantly improved small nerve fibre function in the C57BLKS‐*db/db* murine model of T2DM 20. However, pioglitazone had no effect on the phenotypical measurement of large myelinated fibre function.

In this study, we expand on our previous findings by evaluating gene expression changes in both the nerve and kidney from control (*db/+*), diabetic (*db/db*) and pioglitazone‐treated (*db/+* PIO and *db/db* PIO) mice using RNA‐sequencing (RNA‐Seq); we subsequently analyse these changes using both differential analysis 20 and self‐organizing maps (SOMs) 21, 22, 23. This combination of analyses, tissues and treatment represents several important advances over previous studies examining diabetes and pioglitazone. First, RNA‐Seq provides more complete transcriptomic information than microarray analysis and is much more sensitive and specific 24. Second, we expand our tissue analysis to include the kidney which provides key information into the mechanisms of DN as well as pioglitazone treatment. Third, the simultaneous analysis and subsequent comparison of both nervous and renal tissue allows us to assess the tissue‐specific effects of pioglitazone as well as the basic mechanisms underlying diabetic complications in peripheral tissue. Finally, the parallel use of two forms of RNA‐Seq analysis will alleviate the wide variety of results that can be generated using common software packages 25, 26.

## Materials and methods

### Animals

**Figure 1:**
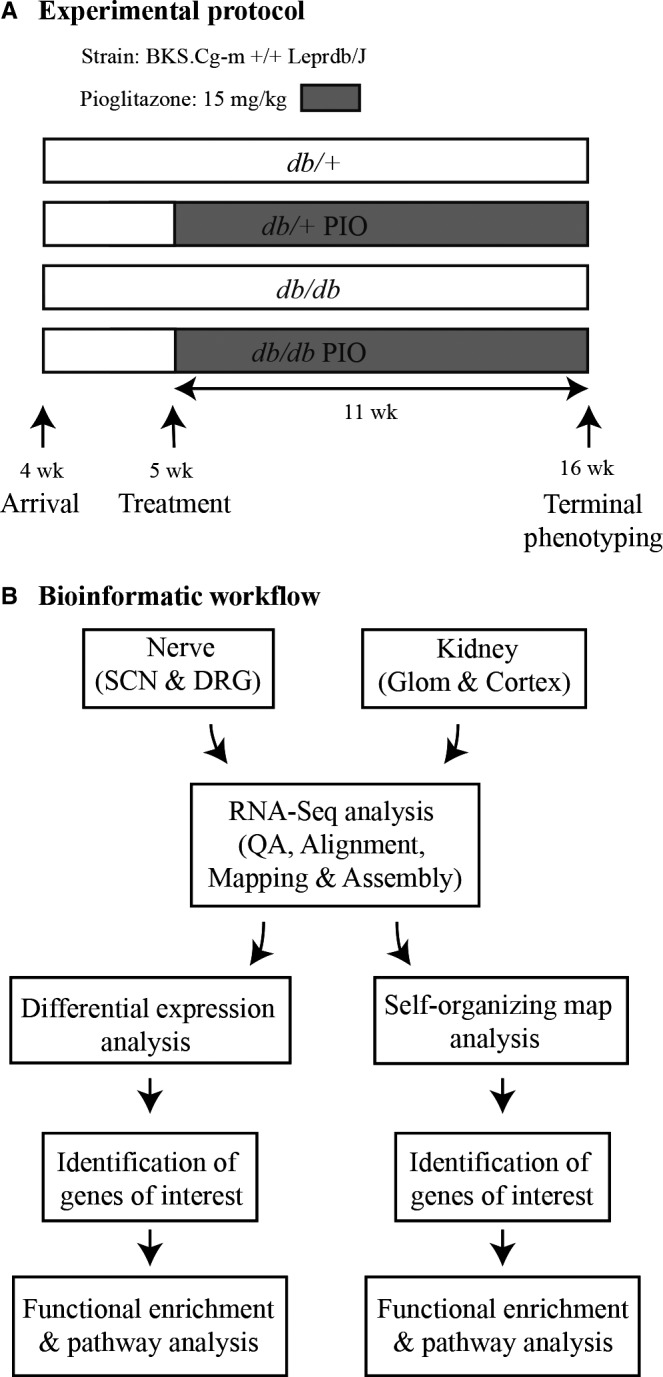
Study workflow. (**A**) *db/+* and *db/db* mice were treated with or without 15 mg/kg pioglitazone (112.5 mg pioglitazone/kg chow, for a final dose of 15 mg/kg to the mouse) from 5 to 16 week of age. (**B**) Total RNA from nerve and kidney tissues was isolated for RNA‐Seq analysis. RNA‐Seq data were mapped, aligned and used for differential expression and self‐organizing map analysis. The identified genes of interest were used for functional enrichment analysis. SCN, sciatic nerve; DRG, dorsal root ganglia; Glom, glomeruli; QA, quality assessment.

Male C57BLKS (BKS) *db/+* and *db/db* mice (BKS.Cg‐m+/+Lepr^db^/J; stock number 000642; Jackson Laboratory, Bar Harbor, ME) were fed a standard diet (AIN76A; 11.5% kcal fat; Research Diets, New Brunswick, NJ, USA) and cared for in a pathogen‐free environment by the University of Michigan Unit for Laboratory Animal Medicine. Mice were treated with or without 15 mg/kg pioglitazone (112.5 mg pioglitazone/kg chow for a dose of 15 mg/kg to the mouse) between 5 and 16 weeks of age, for 11 total weeks (Fig. 1A). Animal protocols were approved by the University of Michigan University Committee on Use and Care of Animals and complied with Diabetic Complications Consortium guidelines (https://www.diacomp.org/shared/protocols.aspx).

### Metabolic phenotyping

For each animal, bodyweight was recorded and fasting blood glucose (FBG) levels were measured with an AlphaTrak Glucometer (Abbott Laboratories, Abbott Park, IL, USA) weekly. Glycated haemoglobin (GHb) levels were determined using a Glyco‐Tek Affinity column (catalog no. 5351; Helena Laboratories, Beaumont, TX, USA) at the Michigan Diabetes Research and Training Center Chemistry Core. Fasting plasma insulin, total cholesterol and total triglycerides were measured by the National Mouse Metabolic Phenotyping Center (Vanderbilt University, Nashville, TN, USA).

### DPN and DN phenotyping

All animals were phenotyped for DPN and DN according to Diabetic Complications Consortium guidelines 27, 28. Motor (sciatic) and sensory (sural) nerve conduction velocities (NCVs) were measured for large nerve fibre function, and hind paw withdrawal latency from a thermal stimulus was measured for small fibre function using our published protocols 29, 30. The periodic acid–Schiff (PAS) staining on 3‐μm‐thick fixed kidney slices determined mesangial area as previously described 31, 32. Urinary albumin levels, albumin/creatinine ratios, glomerular area and glomerular PAS‐positive area were measured using our published protocols 31, 32.

### RNA‐Seq

To identify mechanisms affected by pioglitazone in DPN and DN at the transcriptomic level, we analysed steady‐state gene expression using RNA‐Seq (Fig. 1B). Total RNA was isolated from sciatic nerve (SCN), dorsal root ganglia (DRG), and kidney glomeruli (Glom) and cortex from *db/+* (*n* = 6), *db/db* (*n* = 6), *db/+* PIO (*n* = 6), and *db/db* PIO (*n* = 6) mice. RNA quality was assessed using TapeStation (Agilent, Santa Clara, CA). Samples with RNA integrity numbers ≥8 were prepared using the Illumina TruSeq mRNA Sample Prep v2 kit (Catalog #s RS‐122‐2001, RS‐122‐2002; Illumina, San Diego, CA, USA). Multiplex amplification was used to prepare cDNA with a paired‐end read length of 100 bases using an Illumina HiSeq 2000 (Illumina, Inc., San Diego, CA, USA). RNA‐sequencing was performed by the University of Michigan DNA Sequencing Core (http://seqcore.brcf.med.umich.edu/).

Quality control assessment of RNA‐Seq data was completed using the FastQC tool (http://www.bioinformatics.babraham.ac.uk/projects/fastqc/) for high throughput sequencing before and after RNA‐Seq alignment. Then, RNA‐Seq data were analysed using the Tuxedo suite of sequence analysis programs, including Bowtie, TopHat and Cufflinks 33. Using TopHat, the resulting FASTQ files were aligned to the NCBI reference mouse transcriptome (NCBI 37) to identify known transcripts. Mapped reads were processed using the Cufflinks algorithm to calculate fragments per kilobase of exon per million mapped reads (FPKM), which accurately reflects the RNA transcript number normalized for RNA length and total number of mapped reads 33.

### Differential expression analysis

The output of Cufflinks was loaded into Cuffdiff 33 to quantify differences in expression of combined transcripts for each gene between the groups within each tissue (*db/+ versus db/db*,* db/+ versus db/+* PIO, *db/+* PIO *versus db/db* PIO, and *db/db versus db/db* PIO). The differentially expressed genes (DEGs) with a false discovery rate (FDR) cut‐off of <0.05 were identified between groups, and sets were compared within and across tissues to identify gene expression changes. Analyses focused on *db/+ versus db/db* and *db/db versus db/db* PIO DEG sets to identify gene expression changes in *db/db* mice that were reversed, exacerbated or unaffected by pioglitazone treatment.

### SOM analysis

SOM analysis was performed to identify gene clusters with similar expression patterns in kidney and nerve of *db/+*,* db/db* and *db/db* PIO mice. SOMs generate a two‐dimensional grid and cluster similar patterns of data points into units called modules. FPKM were pre‐processed by removing genes with expression values less than log_2_3 and were centred at zero for each gene 34. Pre‐processed FPKM were applied to a SOM using the algorithm implemented in the MATLAB software Neural Networking Toolbox [www.mathworks.com]. Gene sets having a similar expression pattern were grouped into modules. Each module in the SOM panel was subjected to functional enrichment analysis. Adjacent modules were further combined into clusters that share enriched functions of interest and similar gene expression patterns.

### Function and pathway enrichment analysis

Over‐represented biological functions from the DEG sets and SOM modules were identified by functional enrichment analysis using the Database for Annotation, Visualization and Integrated Discovery (DAVID 6.7) (http://david.abcc.ncifcrf.gov). Gene Ontology terms and Kyoto Encyclopedia of Genes and Genomes pathways were adopted as the functional terms 35. A Benjamini–Hochberg corrected *P*‐value <0.05 was used to identify significantly over‐represented biological functions in the DEG sets. To visualize results, heat maps were generated using the most over‐represented biological functions for DEG sets of interest. Hierarchical clustering based on significance values was used to represent overall similarity and differences between the DEG sets 36. Moreover, clusters from SOM analysis were investigated to identify canonical pathways using Ingenuity Pathway Analysis software (IPA, www.qiagen.com/ingenuity). A Benjamini–Hochberg adjusted *P*‐value was calculated using the Fisher's exact test and <0.05 used to identify significantly over‐represented canonical pathways.

### RNA‐Seq qPCR validation

Technical validation of RNA‐Seq data was performed on glomerular tissue by quantitative real‐time polymerase chain reaction (RT‐qPCR; *n* = 6/group). Biological confirmation was performed on glomerular and SCN tissue (*n* = 6/group). We focused on the SOM cluster, containing genes regulated by diabetes, but reversed by pioglitazone treatment in both kidney glomeruli and SCN (Table 1; Fig. 4) as this cluster likely represents pathways that may drive both DN and small nerve fibre dysfunction and provides insight into conserved pathways in the diabetic kidney and nerve. Our selection of specific genes for RT‐qPCR was based on a combination of expression level (FPKM), fold‐change, FDR significance, *P*‐values (Data S1). Based on our understanding of mitochondrial substrate metabolism in complications‐prone tissue 37, 38, 39, and the established role of oxidative stress in diabetic complications 40, we chose two targets encoding components of fatty acid β‐oxidation (*Acaa2*, and *Echs1* encoding the second and last enzymes of β‐oxidation) for technical validation, and two targets encoding subunits of complex II and complex IV of the mitochondrial electron transport system (*Sdhb*, complex II; *Cox4i1*, complex IV), and a target encoding a mitochondrial peroxynitrite antioxidant enzyme, peroxiredoxin‐5 (*Prdx5*) for biological confirmation. cDNA was generated from 40 ng of total RNA (iScript cDNA Synthesis Kit; Bio‐Rad, Hercules, CA). RT‐qPCR was performed in triplicate using sequence‐specific primers (Table S16), Power SYBR^®^ Green PCR Master Mix (Applied Biosystems/Waltham, MA, USA) and the StepOnePlus™ Real‐Time PCR System (Applied Biosystems/Waltham, MA, USA). Expression of each gene was calculated from a cDNA titration within each plate (standard curve method) and normalized to the geometric mean of tyrosine 3‐monooxygenase/tryptophan 5‐monooxygenase activation protein (*Ywhaz*) endogenous reference gene expression. Samples were assayed in triplicate.

**Table 1:** Pathway enrichment analysis of SOM Cluster. Top 20 significantly enriched canonical pathways among the shared genes in modules 42 and 49 from the SOM analysis using IPA^a^

Canonical pathways	BH *P*‐value^a^	Genes
Mitochondrial dysfunction	7.94E−20	*Ndufa4, Sdhb, Cox7b, Cox6a1, Cox6c, Prdx5, Uqcr11, Xdh, Aco2, Ndufb3, Ndufb10, Pdha1, Ndufb9, Ndufab1, Ndufb6, Aco1, Atp5 g3, Cox4i1, Sdha, Ndufv1, Cox6b1, Ndufb4, Cycs, Ndufv3, Uqcrb, Gsr, Atp5b, Uqcr10, Uqcrc2, Cyc1, Cox5a, Cox7a2, Ndufa12, Atpaf2, Uqcrq*
Oxidative phosphorylation	7.94E−20	*Ndufa4, Sdhb, Cox7b, Cox6a1, Cox6c, Uqcr11, Ndufb3, Ndufb10, Ndufb9, Ndufab1, Ndufb6, Atp5 g3, Cox4i1, Sdha, Ndufv1, Cox6b1, Ndufb4, Cycs, Ndufv3, Uqcrb, Atp5b, Uqcr10, Uqcrc2, Cyc1, Cox5a, Cox7a2, Ndufa12, Atpaf2, Uqcrq*
TCA cycle II (Eukaryotic)	3.16E−17	*Sdha, Sdhb, Idh3 g, Aco2, Mdh1, Sucla2, Cs, Suclg1, Dlst, Dld, Idh3a, Mdh2, Fh, Aco1, Idh3b*
Glycolysis I	4.37E−07	*Pgk1, Eno1, Tpi1, Pgam1, Pkm, Aldoa, Gapdh, Pfkl, Aldoc*
Glutaryl‐CoA degradation	6.17E−06	*Hadhb, L3hypdh, Acat1, Ehhadh, Hsd17b4, Hadh*
Gluconeogenesis I	6.17E−06	*Pgk1, Eno1, Pgam1, Aldoa, Gapdh, Mdh1, Mdh2, Aldoc*
Valine degradation I	7.41E−06	*Hadhb, Echs1, Bcat2, Bckdha, Dld, Dbt, Ehhadh*
Acetyl‐CoA Biosynthesis I (Pyruvate dehydrogenase complex)	8.71E−06	*Pdha1, Dlat, Dld, Dbt, Pdhb*
Fatty acid β‐oxidation I	2.00E−05	*Hadhb, Echs1, Ehhadh, Hsd17b4, Acadm, Acaa2, Eci1, Hadh*
Isoleucine degradation I	2.14E−05	*Hadhb, Echs1, Bcat2, Acat1, Dld, Ehhadh*
Tryptophan degradation III (eukaryotic)	2.19E−04	*Hadhb, L3hypdh, Acat1, Ehhadh, Hsd17b4, Hadh*
Sucrose degradation V (Mammalian)	1.32E−03	*Tpi1, Aldoa, Galm, Aldoc*
Branched‐chain α‐keto acid dehydrogenase complex	1.70E−03	*Bckdha, Dld, Dbt*
Pentose phosphate pathway (oxidative branch)	3.89E−03	*Pgd, Pgls, G6pd*
Lipoate biosynthesis and incorporation II	1.55E−02	*Lipt1, Lias*
Leucine degradation I	2.69E−02	*Bcat2, Acadm, Mccc2*
Ascorbate recycling (cytosolic)	3.55E−02	*Glrx, Gsto1*
Glutathione redox reactions II	3.55E−02	*Gsr, Glrx*
Fatty acid β‐oxidation III (unsaturated, odd number)	3.55E−02	*Ehhadh, Eci1*
Pentose phosphate pathway	4.07E−02	*Pgd, Pgls, G6pd*

a IPA: ingenuity pathway analysis; BH *P*‐value: Benjamini–Hochberg *P*‐value.

### Statistical analyses of phenotypic data

Statistical analyses of phenotypic and RT‐qPCR data utilized GraphPad Prism Software, version 6 (GraphPad Software, La Jolla, CA, USA). Data were assumed to follow a Gaussian distribution based on the rules for transformation and non‐normative data 41. One‐way ANOVA with Tukey's post‐test for multiple comparisons or Kruskal–Wallis test with Dunn's post‐test for multiple comparisons was used, as appropriate 42. The correlation matrix was generated from Pearson's correlations. Data were considered significant when *P* < 0.05. Reported values represent the mean ± SEM.

## Results

### Bioinformatic workflow and confirmation of pioglitazone efficacy in the kidney and small nerve fibres

To determine the differential effects of pioglitazone on DPN and DN and to elucidate potential mechanisms explaining tissue‐specific differences, we compared the metabolic, neurological and renal phenotypes of *db/+* and *db/db* mice with and without pioglitazone treatment (Fig. 1A). We next identified differentially regulated cellular pathways using differential analysis and SOMs to analyse RNA transcripts from the SCN, DRG, kidney glomeruli and kidney cortex (Fig. 1B). Consistent with our previous studies 20, *db/db* mice receiving pioglitazone treatment were significantly heavier than both *db/+* and *db/db* mice, but had significantly reduced blood glucose levels and GHb % with no significant effect on insulin, cholesterol or triglyceride levels (Fig. S1). Also consistent with our previous study, we found that pioglitazone could significantly prevent small nerve fibre dysfunction, but large nerve fibre dysfunction was unaffected by treatment (Fig. S2). In contrast, pioglitazone had a significant effect on DN anatomical and physiological markers of renal function (Figs S3 and S4). Due to the positive effect of pioglitazone treatment on hyperglycaemia, small fibre dysfunction (hind paw thermal latency) and DN, we performed correlation analyses between these parameters (Fig. S5; Data S1). All correlations were significant, suggesting a close relationship between glycemia, small fibre dysfunction and DN. Taken together, these data indicate that pioglitazone treatment selectively affects different aspects of metabolism and functions in a tissue‐specific manner during T2DM.

### Differential expression analysis of tissue‐specific RNA transcripts identifies reversed and exacerbated genes associated with DPN, DN and pioglitazone treatment

**Figure 2:**
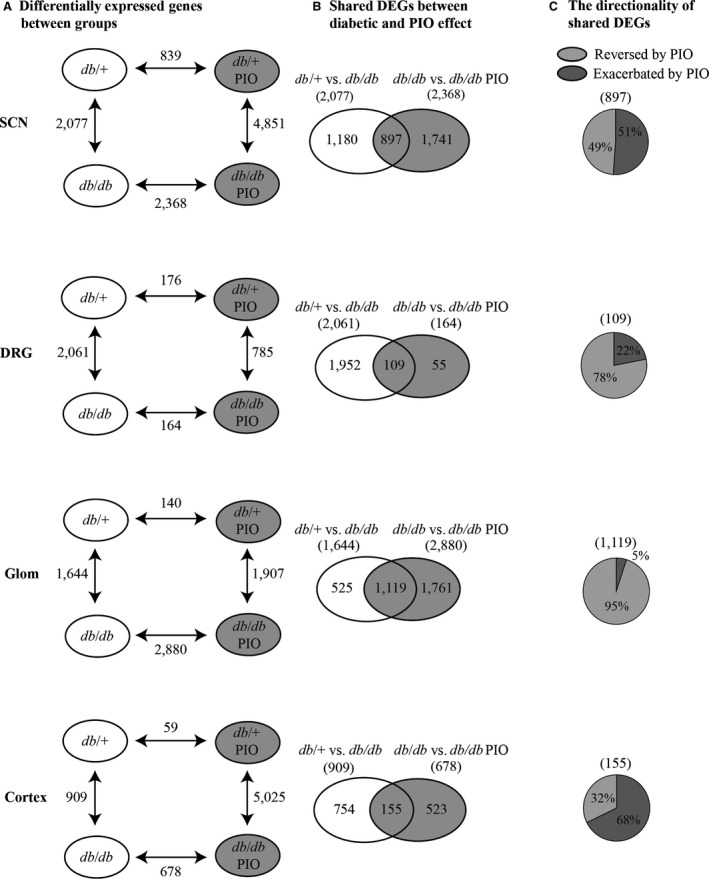
Differential expression analysis. RNA‐Seq data were used to determine gene expression in nerve (SCN, DRG) and kidney (Glom, cortex) tissues from all groups. (**A**) Differential gene expression analysis was determined using Cuffdiff with a false discovery rate (FDR) cut‐off of <0.05. Pairwise comparisons were performed between DEG sets for all groups within a tissue. DEGs regulated by both diabetes and pioglitazone within a tissue were determined (*db/+ versus db/db* and *db/db versus db/db* PIO). Venn diagrams illustrate the shared and unique DEGs between the two groups. (**B**) Directionality of regulation of these overlapping DEG sets was assessed, and the shared genes were divided into two groups: DEGs Reversed by PIO and DEGs Exacerbated by PIO. (**C**) The percentage of shared DEGs exacerbated and reversed by PIO is indicated in the pie chart for each tissue. SCN, sciatic nerve; DRG, dorsal root ganglia; Glom, glomeruli.

To identify specific mechanisms differentially affected by pioglitazone in DPN and DN at the transcriptomic level, we first analysed steady‐state gene expression in the SCN, DRG, kidney glomeruli and kidney cortex using RNA‐Seq. This analysis resulted in an average of 29.8 (±8.4) million reads, and the resulting data were subsequently analysed using differential expression analysis (Fig. 1B; Table S1). For each type of tissue, four DEG sets were obtained from the pairwise comparisons (*db/+ versus db/db*,* db/+ versus db/+* PIO, *db/+* PIO *versus db/db* PIO, and *db/db versus db/db* PIO) (Fig. 2A). The number of genes regulated by diabetes (*db/+ versus db/db*) was similar in the SCN (2077) and the DRG (2061); however, pioglitazone significantly changed gene expression in 14‐fold more genes in the diabetic SCN (2368) than in the DRG (164). Similarly, in the kidney, the number of DEGs was greater in diabetic glomeruli (1644) than in cortex (909), and pioglitazone changed the expression of fourfold more genes in the diabetic glomeruli (2880) than the cortex (678). These data indicate that even within similar tissue, pioglitazone treatment can have differing effects.

To better understand the cellular mechanisms driving DN and DPN changes in response to pioglitazone treatment, we next compared *db/+ versus db/db* and *db/db versus db/db* pioglitazone DEG sets in each tissue (Fig. 2B). Overall, 897 (43%) SCN DEGs and 1119 (68%) glomeruli DEGs were significantly affected by both diabetes and pioglitazone in *db/db* mice (Tables S2–S9). However, only 109 (5%) DRG and 155 (17%) kidney cortex DEGs were affected by treatment. To address the discrepancy, we examined the transcript expression of *Ppara*,* Ppard* and *Pparg* in the SCN, DRG, kidney glomeruli and kidney cortex (Fig. S6). Pioglitazone is a PPARG agonist; therefore, we suggested that the low number of genes regulated by pioglitazone treatment in DRG and cortex could be due to low PPAR expression in these tissue types. Overall, the number of *Ppar* transcripts was highest in kidney glomeruli and SCN with reduced expression in the kidney cortex and negligible transcript expression in the DRG. Hence, the reduced *Ppar* expression in the DRG and the kidney cortex likely explains the relatively low numbers of shared DEGs in these tissues. Our subsequent analyses therefore focused primarily on the SCN and glomeruli, as these tissues are affected by pioglitazone treatment.

We next determined whether DEGs shared between the *db/+ versus db/db*, and *db/db versus db/db* PIO DEG sets were regulated in the opposite (reversed) or same (exacerbated) direction (Fig. 2C). As large fibre dysfunction is unaffected by pioglitazone treatment in *db/db* mice (Fig. S2), we reasoned that DEGs in SCN that are significantly up‐regulated during diabetes but not reversed by pioglitazone treatment (Table S2) may contribute to large fibre dysfunction. In contrast, genes reversed by pioglitazone treatment in the SCN (Table S7) and reversed in the glomeruli (Table S8) may prevent damage of the small nerve fibres and the kidney during T2DM. Consistent with our phenotypic data, only half of the shared DEGs in SCN (49%) were reversed by pioglitazone treatment while the majority of the shared DEGs in glomeruli (95%) were reversed. These data suggest that pioglitazone may contribute to large nerve fibre dysfunction by exacerbating a tissue‐specific subset of genes within the SCN while ameliorating DN *via* a completely different mechanism.

### Comparison of pathways using differential expression analysis identifies cellular pathways associated with tissue‐specific pioglitazone function

**Figure 3:**
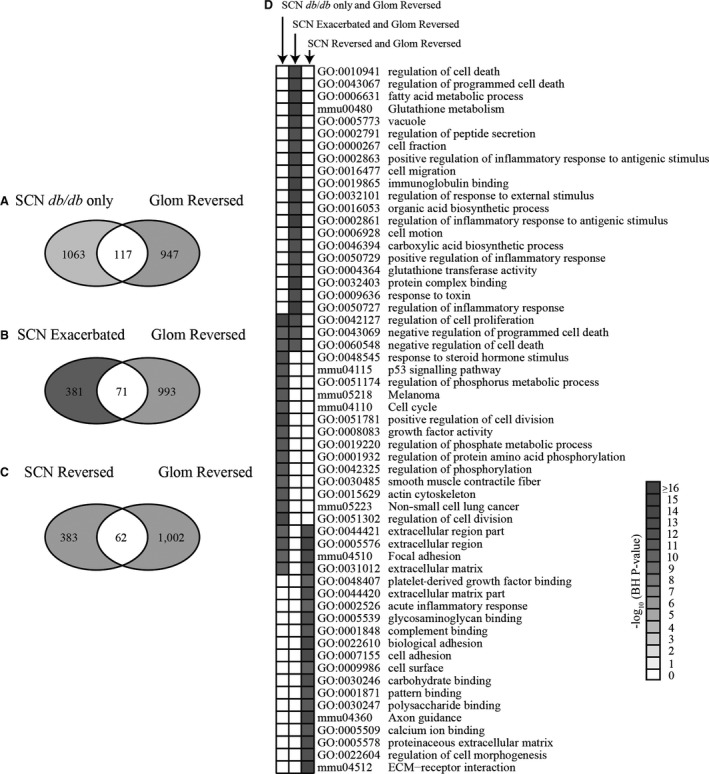
Analysis of DEGs between SCN and glomeruli. The DEG sets were analysed between the DEGs reversed by pioglitazone treatment in glomeruli and three groups of DEGs in SCN: (**A**) SCN *db/db* only, (**B**) SCN Exacerbated and (**C**) SCN Reversed. (**D**) DAVID functional enrichment analysis was performed on the shared DEGs from each comparison. Over‐represented functions are shown in the heat map with *P*‐value <0.05.

We next used the DEGs found in the SCN and kidney glomeruli to determine which cellular pathways are associated with DPN, DN and pioglitazone treatment. DN phenotypes were completely prevented by pioglitazone (Fig. S3), while the effects of pioglitazone on DPN were limited to small fibre function (Figs S1 and S2). Therefore, to identify unique pathways underlying these tissue‐specific differences, we compared the three DEG subsets from SCN that were either (A) not affected by pioglitazone in diabetic mice (SCN *db/db* only), (B) exacerbated by pioglitazone (SCN Exacerbated) or (C) reversed by pioglitazone (SCN Reversed), to DEGs reversed by pioglitazone in the kidney glomeruli (Glom Reversed; Fig. 3A‐C). This was done to identify pathways associated with large fibre dysfunction, small fibre dysfunction and DN. DEGs shared between the SCN *db/db* only and the Glom Reversed sets are genes that may drive large fibre dysfunction (Fig. 3A); there were a total of 117 DEGs shared between these two data sets. DEGs shared between the SCN Exacerbated and the Glom Reversed data sets indicate genes that may drive both DPN and DN but are not reversed in the SCN by pioglitazone (Fig. 3B); there were 71 shared DEGs in the data sets. Finally, shared DEGs between the SCN Reversed and the Glom Reversed data sets indicate genes that are reversed in both tissues by pioglitazone. As small fibre dysfunction is prevented by pioglitazone, overlapping DEGs in this data set may therefore contribute to small fibre dysfunction (Fig. 3C). The SCN Reversed and Glom Reversed data set was comprised of 62 DEGs; the top 20 up‐ and down‐regulated shared DEGs for each of the three comparisons are listed in Tables S10–S15.

In both the SCN *db/db* only *versus* Glom Reversed (Fig. 3A) and SCN Reversed *versus* Glom Reversed (Fig. 3C) DEG comparisons, our functional analysis using DAVID identified enriched pathways related to extracellular matrix (ECM) remodelling and focal adhesion (Fig. 3D). In the SCN *db/db* only *versus* Glom Reversed data sets, we found gene expression changes in collagen, type I, alpha 1 (*Col1a1*), SRC kinase signalling inhibitor 1 (*Srcin1*) and *Spon2*, suggesting that these DEGs may be involved in DN and large fibre dysfunction in DPN (Table S11). In contrast, pioglitazone reversed expression of several DEGs in both the SCN and the kidney glomeruli (SCN Reversed *versus* Glom Reversed). Bone morphogenetic protein 3 (*Bmp3*), laminin, gamma 2 (*Lamc2*), type VI, alpha 1 collagen (*Col6a1*), and type III, alpha 1 collagen (*Col3a1*) were reversed in both tissue types suggesting that the associated pathways may be involved in DN and DPN small fibre dysfunction (Table S15). Together, these data suggest that correcting changes related to tissue remodelling in the glomeruli has a large impact on DN, but the associated pathways have a more complex relationship with regard to the SCN and DPN.

Of particular interest were the differential effects of pioglitazone in nerve and kidney seen in the SCN *db/db* only *versus* Glom Reversed (Fig. 3A) and SCN Exacerbated *versus* Glom Reversed comparisons (Fig. 3B; Tables S10‐S13), as these pathways may contribute to the pathogenesis of both DPN and DN (pioglitazone treatment had no effect on large fibre DPN but reversed DN). Among the shared DEGs, there was functional enrichment of multiple categories related to cell death and the inflammatory response (Fig. 3D).

### Identification of dysregulated molecular pathways associated with pioglitazone treatment in the SCN and glomeruli using self‐organizing map analysis

**Figure 4:**
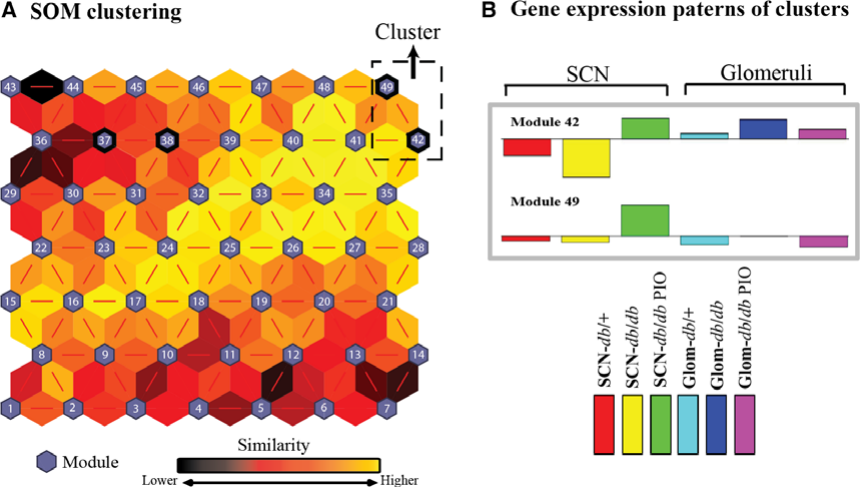
Analysis of self‐organizing maps. SOM analysis was applied to the RNA‐Seq data to identify coherent patterns of gene expression across six groups: *db/+*,* db/db* and *db/db* PIO in SCN and glomeruli. (**A**) SOM clustering analysis demonstrates the distances between correlated gene groups. Small blue hexagons represent a module containing genes with a similar expression pattern. The neighbouring modules are connected with a red line. The colours between the modules indicate the similarity between modules: Lighter colours represent higher similarity, and darker colours represent lower similarity. (**B**) Gene expression patterns of biological interest were identified, and a cluster comprised of modules 42 and 49 was further analysed.

Previous studies have shown that the use of differential expression analysis for analysing RNA transcripts can produce very different results depending on a number of factors 25. To support the results generated using differential expression analysis, we utilized SOM analysis to identify similar patterns of gene expression across the three experimental groups (*db/+*,* db/db* and *db/db* PIO) in both the SCN and the kidney glomeruli. After removal of very low expression values, 15,588 genes remained for SOM analysis. Genes with similar expression patterns were grouped into modules and plotted as a 7 × 7 map to empirically identify biologically meaningful pathways (Fig. 4A). Genes with the most variation across the experimental groups are gathered in the modules in the top left and bottom right corners of the grid map, whereas genes with less variation across groups are gathered around the centre of the map. As a screen to identify modules of interest, we performed DAVID for all 49 modules and determined the most over‐represented biological functions (Fig. S7). By combining adjacent modules with similar expression patterns, we were able to define functional clusters of interest and identify pathways associated with diabetic complications and pioglitazone treatment (Table 1; Fig. 4B).

The regulation pattern of genes in modules 42 and 49 is analogous to the differential expression analysis of genes in the SCN Reversed and Glom Reversed groups and represents conserved pathways that may drive both DN and small nerve fibre dysfunction (Fig. 3C). This cluster contained pathways related to mitochondrial dysfunction, oxidative phosphorylation, glycolysis, fatty acid β‐oxidation and the TCA cycle (Table 1). Genes of interest that were reversed included those encoding subunits of the mitochondrial complexes (complex I NADH oxidoreductase, *Ndufa4/12*,* Ndufb3/4/6//9/10*,* Ndufv1/3*; complex II, *Sdha*,* Sdhb*; complex IV, *Cox4i1*,* Cox5a*,* Cox6a1*,* Cox6b1*,* Cox6c*,* Cox7a2*,* Cox7b*; and complex V, *Atp5g3*,* Atp5b*,* Atpaf2*), and β‐oxidation enzymes (*Acaa2*,* Echs1*). Notably, although these pathways are conserved across the tissues, they are largely opposite in their directionality of regulation (Fig. 4B). Selected genes in the cluster were validated in SCN and glomeruli using RT‐qPCR, which demonstrated comparable profiles to the RNA‐Seq data (Table S16). Collectively, these observations highlight tissue‐specific pathways associated not only with diabetes pathogenesis but with pioglitazone treatment.

## Discussion

Available treatments for DPN and DN can have variable efficacy in small nerve fibres, large nerve fibres and kidneys, suggesting that tissue‐specific mechanisms occur in response to treatment. We recently reported that pioglitazone, a triglyceride‐lowering, insulin‐sensitizing PPARG agonist, has differing effects on DPN and DN phenotypes in a mouse model of diabetes 20. The goal of this study was therefore to elucidate the shared and unique mechanisms underlying DN and DPN in response to pioglitazone treatment. Using the same experimental paradigm as our previous study, we confirmed our previous observations that pioglitazone prevents small nerve fibre and renal dysfunction but is unable to prevent large nerve fibre dysfunction during DPN. We then used RNA‐Seq combined with a combination of differential expression analysis and SOM analysis to determine molecular pathways that may be driving tissue‐specific differences.

We evaluated gene expression changes in the nerve and kidney of control (*db/+*), diabetic (*db/db*) and pioglitazone‐treated (*db/+* PIO and *db/db* PIO) mice. Differential expression analysis showed that pioglitazone had a greater effect on SCN and kidney glomeruli gene expression than on DRG and cortex profiles, likely due to reduced PPAR expression in the DRG and the kidney cortex. Subsequent analysis therefore focused on SCN and kidney glomeruli. Consistent with the phenotypic data, in the SCN, 897 shared genes were regulated by both diabetes and pioglitazone, with approximately half of the overlapping genes exacerbated and half reversed by pioglitazone. Those reversed by pioglitazone likely contribute to the prevention of small fibre dysfunction, while those exacerbated or unaffected by pioglitazone likely contribute to large fibre dysfunction. In contrast, of the 1119 shared genes altered in the kidney glomeruli during diabetes, virtually all (95%) were reversed by pioglitazone treatment.

As small fibre dysfunction and DN correlated strongly with glycemia (Fig. S5), gene expression reversal may be a downstream effect of preventing hyperglycaemia (Fig. S1). It is unclear to what extent the changes seen following pioglitazone treatment are due to direct PPAR inhibition or prevention of hyperglycaemia. Therefore, while it is obvious that exacerbated changes in the large nerve fibre are directly due to pioglitazone treatment, the prevention of DN and small nerve fibre dysfunction may be partially due to prevention of hyperglycaemia. The enhanced expression of *Ppar* isoforms in tissue with high numbers of DEGs (Fig. S6), however, suggests that *Ppar* inhibition plays a key role in the observed changes. Further studies will be needed to determine the direct impact of systemic metabolic changes on gene expression in the nerve and kidney.

Consistent with previous reports, genes associated with tissue remodelling such as *Grem1*,* Grem2* and *Spon2* were significantly up‐regulated in the kidney glomeruli during diabetes but reversed by pioglitazone (Table S8) 43, 44. Similarly, levels of SPON2, an ECM protein involved in innate immunity, correlate with DN severity in T2DM patients 45; we observed increased *Spon2* levels in the diabetic kidney that were reversed by pioglitazone treatment. Our data therefore suggest that changes in tissue remodelling and ECM function within kidney glomeruli are involved in DN pathophysiology but ameliorated by pioglitazone. Many of these pathways are unaffected or even up‐regulated in the presence of pioglitazone in the SCN, however. For example, *Col1a1*,* Srcin1* and *Spon2* are reversed in glomeruli, but are unaffected by pioglitazone in SCN, suggesting a role for these genes in large fibre dysfunction (Table S11).

In contrast, expression of other genes associated with tissue remodelling such as *Bmp3*,* Lamc2*,* Col6a1* and *Col3a1* was reversed in both the kidney glomeruli and the SCN in response to pioglitazone (Table S15). This suggests a role for these genes in small fibre dysfunction. Indeed, injection of *Bmp2*‐overexpressing fibroblasts can promote sensory nerve remodelling and neurogenic inflammation in C57BL/6 mice 46. Regardless of large/small fibre stratification, these data implicate dysfunctional ECM signalling and tissue remodelling as shared pathogenic mechanisms between DN and DPN.

Inflammatory pathways are also differentially regulated in DPN and DN. *Mmp12*, part of the inflammatory matrix metalloproteinase family, was up‐regulated in the SCN 143‐fold during diabetes but unaffected by pioglitazone treatment (Table S2), supporting our previous study which demonstrated *Mmp12* up‐regulation in the SCN of leptin‐deficient BTBR *ob/ob* mice 36. In contrast, *Mmp12* deletion in diabetic mice reduces kidney glomeruli matrix accumulation and markers of inflammation, suggesting an important but reversible role for MMP12 in driving kidney complications 47.

To confirm these results, we also used SOM analysis to detect tissue‐specific transcription changes in the SCN and kidney glomeruli following pioglitazone treatment. We focused on modules 42 and 49 as their shared pattern of gene expression compares genes that are reversed by pioglitazone treatment in both the kidney glomeruli and SCN (analogous to the Glom Reversed and SCN Reversed differential expression analysis in Fig. 3C). This pattern offers mechanistic insight into conserved pathways that may drive both DN and small nerve fibre dysfunction in T2DM. Moreover, this gene cluster likely has greater translational relevance as DPN is predominantly a small fibre disease 6.

This SOM cluster showed enriched transcripts related to mitochondrial dysfunction, fatty acid β‐oxidation, the TCA cycle and oxidative phosphorylation (Table 1). These data support our previous transcriptomics finding that SCN energy homeostasis is important in small fibre neuropathy 20. Indeed, regulation of these transcripts during diabetic complications is consistent with previous reports demonstrating an up‐regulation of endothelial mitochondrial metabolism in response to excess substrate 48. However, the opposite directionality of change (down‐regulation and reversal in SCN, up‐regulation and reversal in Glom) suggests a more complex relationship with regard to substrate metabolism in diabetic complications‐prone tissues. We recognize that additional mechanistic work is required to explore the biological relevance of transcriptomics data; however, this observation parallels our recent report of tissue‐specific changes in fatty acid flux and mitochondrial metabolism, *in vivo*, in nerve and kidney in BKS‐*db/db* mice 37. Whether these changes in transcriptomics and fluxomics are the cause or the result of diabetes is unknown. Indeed, cross‐complications metabolic reprogramming is the subject of ongoing work by our group.

Lastly, to investigate the reproducibility of our transcriptomic studies, we identified common DEG sets shared between the current RNA‐Seq analysis and our previous microarray DEG study in the SCN and the DRG 20 (Fig. S8A). The number of overlapping DEGs between the studies was relatively low during diabetes (*db/+ versus db/db*: 411 SCN and 241 DRG) and following pioglitazone treatment (*db/db versus db/db* PIO: 1408 SCN and 392 DRG). This may reflect differences in the animal models, the platforms or both. Also, while our data suggest that RNA‐Seq is more sensitive than microarray when detecting DEGs (Fig. S8B), the enriched pathways detected using both techniques were highly similar despite the relatively low number of overlapping DEGs (Fig. S8C).

In summary, the current differential expression and SOM analyses suggest that shared pathogenic mechanisms exist between DPN and DN, including ECM dysfunction, tissue remodelling, inflammation and dysfunctional mitochondrial metabolism. Our data suggest that large fibre dysfunction may be related to inflammation, while mitochondrial metabolism may play a greater role in small fibre pathophysiology in T2DM. Moreover, targeting both of these pathways is likely to improve DN phenotypes. We previously reported that lipid‐targeted, insulin‐sensitizing pioglitazone therapy improved DN, and small fibre measures of DPN. The current study extends those data to suggest that systemic changes in metabolism in T2DM are also associated with distinct tissue‐specific metabolic reprogramming in kidney and nerves (similar pathways regulated, different directionality of regulation) and that these changes play a critical role in DN and small fibre DPN pathogenesis. This new insight highlights the potential dangers of a ‘one size fits all’ approach to T2DM therapeutics, as the same drug may simultaneously alleviate one complication while exacerbating another.

Our analyses therefore have the potential to enhance future treatment of diabetic complications by identifying specific molecular pathways associated with each type of complication.

## Conflict of interest

The authors have no conflict of interests to declare for this work.

## Author's contributions

L.M.H. directed the study, researched data, contributed to discussion and wrote the manuscript. M.P. researched data and wrote the manuscript. A.R. contributed to discussion and wrote the manuscript. J.H. researched data and revised the manuscript. F.E. researched data. M.K. and F.C.B. designed and directed the study, contributed to discussion and wrote the manuscript. S.P contributed to the discussion and wrote the manuscript. E.L.F. designed and directed the study, contributed to discussion and wrote the manuscript. E.L.F. is the guarantor of this work and, as such, had full access to all the data in the study and takes responsibility for the integrity of the data and the accuracy of the data analysis.

## Supporting information


**Table S1** Summary of RNA sequencing.
**Table S2** Top 20 DEGs up‐regulated by diabetes but not affected by pioglitazone treatment in sciatic nerve (SCN *db/db* only in *db/+* vs. *db/db* and *db/db* vs. *db/db* PIO).
**Table S3** Top 20 DEGs down‐regulated by diabetes but not affected by pioglitazone treatment in sciatic nerve (SCN *db/db* only in *db/+* vs. *db/db* and *db/db* vs. *db/db* PIO).
**Table S4** Top 20 DEGs up‐regulated by diabetes but not affected by pioglitazone treatment in glomeruli (Glom *db/db* only in *db/+* vs. *db/db* and *db/db* vs. *db/db* PIO).
**Table S5** Top 20 DEGs down‐regulated by diabetes but not affected by pioglitazone treatment in glomeruli (Glom *db/db* only in *db/+* vs. *db/db* and *db/db* vs. *db/db* PIO).
**Table S6** Top 20 DEGs up‐regulated by diabetes and regulated by pioglitazone treatment in sciatic nerve (*db/+* vs. *db/db* and *db/db* vs. *db/db* PIO).
**Table S7** Top 20 DEGs down‐regulated by diabetes and regulated by pioglitazone treatment in sciatic nerve (*db/+* vs. *db/db* and *db/db* vs. *db/db* PIO).
**Table S8** Top 20 DEGs up‐regulated by diabetes and regulated by pioglitazone treatment in glomeruli (*db/+* vs. *db/db* and *db/db* vs. *db/db* PIO).
**Table S9** Top 20 DEGs down‐regulated by diabetes and regulated by pioglitazone treatment in glomeruli (*db/+* vs. *db/db* and *db/db* vs. *db/db* PIO).
**Table S10** Top 20 shared genes between down‐regulated by diabetes and reversed by pioglitazone treatment in glomeruli and only regulated by diabetes in SCN (down‐regulated Glom Reversed vs. SCN *db/db* only) (sorted by fold‐change of *db/db* vs. *db/db* PIO in glomeruli).
**Table S11** Top 20 shared genes between up‐regulated by diabetes and reversed by pioglitazone treatment in glomeruli and only regulated by diabetes in SCN (up‐regulated Glom Reversed vs. SCN *db/db* only) (sorted by fold‐change of *db/db* vs. *db/db* PIO in glomeruli).
**Table S12** Shared genes between down‐regulated by diabetes and reversed by pioglitazone treatment in glomeruli and exacerbated by pioglitazone treatment in SCN (down‐regulated Glom Reversed vs. SCN exacerbated) (sorted by fold change of *db/db* vs. *db/db* PIO in glomeruli).
**Table S13** Top 20 shared genes between up‐regulated by diabetes and reversed by pioglitazone treatment in glomeruli and exacerbated by pioglitazone treatment in SCN (up‐regulated Glom Reversed vs. SCN exacerbated) (sorted by fold change of *db/db* vs. *db/db* PIO in glomeruli).
**Table S14** Top 20 shared genes between down‐regulated by diabetes and reversed by pioglitazone treatment in glomeruli and reversed by pioglitazone treatment in SCN (down‐regulated Glom Reversed vs. SCN Reversed) (sorted by fold change of *db/db* vs. *db/db* PIO in glomeruli).
**Table S15** Top 20 shared genes between up‐regulated by diabetes and reversed by pioglitazone treatment in glomeruli and reversed by pioglitazone treatment in SCN (up‐regulated Glom Reversed vs. SCN Reversed) (sorted by fold change of *db/db* vs. *db/db* PIO in glomeruli).
**Table S16** Mouse primer sequences and gene expression validation of RNA‐Seq data using RT‐qPCR.
**Fig. S1** Metabolic phenotyping.
**Fig. S2** Neuropathy phenotyping.
**Fig. S3** Nephropathy phenotyping.
**Fig. S4** Effects of pioglitazone on kidney function.
**Fig. S5** Correlation analysis.
**Fig. S6** Log_2_(x + 1) FKPM of PPAR isoforms across tissues.
**Fig. S7** Functional Enrichment Analysis for all 49 modules from SOM analysis.
**Fig. S8** Comparisons of DEG sets from microarray and RNA‐Seq analyses.

**Data S1**: Supplementary data file containing expanded Correlation Matrix, and Cluster 2 gene expression matrix.
