# Supplementary material for: Comparative RNA‐Seq transcriptome analyses reveal distinct metabolic pathways in diabetic nerve and kidney disease
Source: J Cell Mol Med. 2017 Mar 8;21(9):2140–52. doi: 10.1111/jcmm.13136 (PMC5571536; doi:10.1111/jcmm.13136)
Supplement: Supplementary file 1 — Table S1 Summary of RNA sequencing. Table S2 Top 20 DEGs up‐regulated by diabetes but not affected by pioglitazone treatment in sciatic nerve (SCN db/db only in db/+ vs. db/db and db/db vs. db/db PIO). Table S3 Top 20 DEGs down‐regulated by diabetes but not affected by pioglitazone treatment in sciatic nerve (SCN db/db only in db/+ vs. db/db and db/db vs. db/db PIO). Table S4 Top 20 DEGs up‐regulated by diabetes but not affected by pioglitazone treatment in glomeruli (Glom db/db only in db/+ vs. db/db and db/db vs. db/db PIO). Table S5 Top 20 DEGs down‐regulated by diabetes but not affected by pioglitazone treatment in glomeruli (Glom db/db only in db/+ vs. db/db and db/db vs. db/db PIO). Table S6 Top 20 DEGs up‐regulated by diabetes and regulated by pioglitazone treatment in sciatic nerve (db/+ vs. db/db and db/db vs. db/db PIO). Table S7 Top 20 DEGs down‐regulated by diabetes and regulated by pioglitazone treatment in sciatic nerve (db/+ vs. db/db and db/db vs. db/db PIO). Table S8 Top 20 DEGs up‐regulated by diabetes and regulated by pioglitazone treatment in glomeruli (db/+ vs. db/db and db/db vs. db/db PIO). Table S9 Top 20 DEGs down‐regulated by diabetes and regulated by pioglitazone treatment in glomeruli (db/+ vs. db/db and db/db vs. db/db PIO). Table S10 Top 20 shared genes between down‐regulated by diabetes and reversed by pioglitazone treatment in glomeruli and only regulated by diabetes in SCN (down‐regulated Glom Reversed vs. SCN db/db only) (sorted by fold‐change of db/db vs. db/db PIO in glomeruli). Table S11 Top 20 shared genes between up‐regulated by diabetes and reversed by pioglitazone treatment in glomeruli and only regulated by diabetes in SCN (up‐regulated Glom Reversed vs. SCN db/db only) (sorted by fold‐change of db/db vs. db/db PIO in glomeruli). Table S12 Shared genes between down‐regulated by diabetes and reversed by pioglitazone treatment in glomeruli and exacerbated by pioglitazone treatment in SCN (down‐regulated Glom Reversed vs. SCN e [file JCMM-21-2140-s001.docx]

**Supplemental Materials**

**Comparative RNA-Seq Transcriptome Analyses Reveal Distinct Metabolic Pathways in Diabetic Nerve and Kidney Disease**

Lucy M. Hinder^1$^, Meeyoung Park^1$^, Amy E. Rumora^1$^, Junguk Hur^5^, Felix Eichinger^2^, Subramaniam Pennathur^2^, Matthias Kretzler^2,3^, Frank C. Brosius III^2,4^ and Eva L. Feldman^1*^

Departments of ^1^Neurology, ^2^Internal Medicine, ^3^Computational Medicine and Bioinformatics, ^4^Molecular and Integrative Physiology, University of Michigan, Ann Arbor, MI 48109, USA

^5^Department of Biomedical Sciences, University of North Dakota, School of Medicine and Health

Sciences, Grand Forks, ND 58202, USA

^$^These authors contributed equally.

^*^Corresponding author:

Eva L. Feldman, MD, PhD

Russell N. DeJong Professor of Neurology

5017 AAT-BSRB, 109 Zina Pitcher Place

Ann Arbor, Michigan 48109, United States

Phone: (734) 763-7274 / Fax: (734) 763-7275

Email: [efeldman@umich.edu](mailto:efeldman@umich.edu)

**Supplementary Table 1. Summary of RNA Sequencing**

| Tissue | Reads (millions) | Mapped | %Mapped | MultiReads | % MultiReads | % AlignRate |
| --- | --- | --- | --- | --- | --- | --- |
| Overall | 29.8 (8.4) | 23.4 (6.6) | 78.6% (2.0%) | 1.3 (0.5) | 5.4% (1.2%) | 78.6% (2.0%) |
| Cortex | 32.1 (5.2) | 25.3 (4.0) | 79.0% (1.3%) | 1.8 (0.4) | 7.0% (1.5%) | 79.0% (1.3%) |
| DRG | 28.8 (8.0) | 23.2 (6.4) | 80.4% (1.0%) | 1.1 (0.3) | 4.6% (0.3%) | 80.4% (1.0%) |
| Glom | 29.7 (4.6) | 23.0 (3.6) | 77.4% (1.6%) | 1.2 (0.2) | 5.0% (0.3%) | 77.4% (1.6%) |
| SCN | 28.7 (13.0) | 22.2 (10.0) | 77.3% (2.1%) | 1.2 (0.5) | 5.2% (0.4%) | 77.3% (2.1%) |

**Supplementary Table 2. Top 20 DEGs up-regulated by diabetes but not affected by pioglitazone treatment in sciatic nerve (SCN *db/db* only in *db/+* vs. *db/db* and *db/db* vs. *db/db* PIO).**

| GeneID | Symbol | Description | log2 FC | FDR |
| --- | --- | --- | --- | --- |
| 17381 | *Mmp12* | matrix metallopeptidase 12 | 7.15 | 7.72E-04 |
| 14068 | *F7* | coagulation factor VII | 6.54 | 2.00E-02 |
| 216343 | *Tph2* | tryptophan hydroxylase 2 | 4.92 | 7.72E-04 |
| 16411 | *Itgax* | integrin alpha X | 4.82 | 7.72E-04 |
| 76757 | *Trdn* | triadin | 4.51 | 7.72E-04 |
| 16197 | *Il7r* | interleukin 7 receptor | 4.37 | 7.72E-04 |
| 16846 | *Lep* | leptin | 3.64 | 7.72E-04 |
| 75590 | *Dusp9* | dual specificity phosphatase 9 | 3.53 | 2.03E-03 |
| 69585 | *Hfe2* | hemochromatosis type 2 (juvenile) (human homolog) | 3.53 | 1.42E-02 |
| 21393 | *Tcap* | titin-cap | 3.50 | 7.72E-04 |
| 108723 | *Card11* | caspase recruitment domain family, member 11 | 3.46 | 7.72E-04 |
| 13034 | *Ctse* | cathepsin E | 3.40 | 2.60E-02 |
| 432838 | *Gm5460* | predicted gene 5460 | 3.15 | 1.81E-02 |
| 229665 | *Ampd1* | adenosine monophosphate deaminase 1 | 3.08 | 2.00E-02 |
| 20877 | *Aurkb* | aurora kinase B | 3.04 | 6.63E-03 |
| 109648 | *Npy* | neuropeptide Y | 3.03 | 2.10E-02 |
| 18787 | *Serpine1* | serine (or cysteine) peptidase inhibitor, clade E, member 1 | 3.00 | 7.72E-04 |
| 14120 | *Fbp2* | fructose bisphosphatase 2 | 3.00 | 3.32E-02 |
| 16428 | *Itk* | IL2 inducible T cell kinase | 2.99 | 7.72E-04 |
| 237256 | *Zc3h12d* | zinc finger CCCH type containing 12D | 2.98 | 7.72E-04 |

log2 FC: log_2_(fold-change), FDR: false-discovery rate

**Supplementary Table 3. Top 20 DEGs down-regulated by diabetes but not affected by pioglitazone treatment in sciatic nerve (SCN *db/db* only in *db/+* vs. *db/db* and *db/db* vs. *db/db* PIO).**

| GeneID | Symbol | Description | log2 FC | FDR |
| --- | --- | --- | --- | --- |
| 13107 | *Cyp2f2* | cytochrome P450, family 2, subfamily f, polypeptide 2 | -5.64 | 7.72E-04 |
| 18979 | *Pon1* | paraoxonase 1 | -4.56 | 7.72E-04 |
| 326623 | *Tnfsf15* | tumor necrosis factor (ligand) superfamily, member 15 | -3.06 | 7.72E-04 |
| 20538 | *Slc6a2* | solute carrier family 6 (neurotransmitter transporter, noradrenalin), member 2 | -3.00 | 7.72E-04 |
| 11556 | *Adrb3* | adrenergic receptor, beta 3 | -2.76 | 7.72E-04 |
| 71412 | *Dhrs2* | dehydrogenase/reductase member 2 | -2.63 | 7.72E-04 |
| 11602 | *Angpt4* | angiopoietin 4 | -2.57 | 4.21E-03 |
| 53311 | *Mybph* | myosin binding protein H | -2.46 | 8.96E-03 |
| 170786 | *Cd209a* | CD209a antigen | -2.45 | 7.72E-04 |
| 78896 | *1500015O10Rik* | RIKEN cDNA 1500015O10 gene | -2.10 | 5.22E-03 |
| 13035 | *Ctsg* | cathepsin G | -2.09 | 1.38E-02 |
| 384061 | *Fndc5* | fibronectin type III domain containing 5 | -2.04 | 7.72E-04 |
| 22410 | *Wnt10b* | wingless related MMTV integration site 10b | -2.00 | 7.72E-04 |
| 240479 | *Fam69c* | family with sequence similarity 69, member C | -1.95 | 2.06E-02 |
| 72605 | *Car10* | carbonic anhydrase 10 | -1.92 | 2.79E-02 |
| 18782 | *Pla2g2d* | phospholipase A2, group IID | -1.92 | 7.72E-04 |
| 619310 | *Zfp872* | zinc finger protein 872 | -1.91 | 2.31E-02 |
| 76815 | *Calcoco2* | calcium binding and coiled-coil domain 2 | -1.91 | 3.70E-03 |
| 276891 | *Timd4* | T cell immunoglobulin and mucin domain containing 4 | -1.90 | 2.20E-02 |
| 12970 | *Crygs* | crystallin, gamma S | -1.86 | 3.16E-03 |

log2 FC: log_2_(fold-change), FDR: false-discovery rate

**Supplementary Table 4. Top 20 DEGs up-regulated by diabetes but not affected by pioglitazone treatment in glomeruli (Glom *db/db* only in *db/+* vs. *db/db* and *db/db* vs. *db/db* PIO).**

| GeneID | Symbol | Description | log2 FC | FDR |
| --- | --- | --- | --- | --- |
| 381413 | *Gpr176* | G protein-coupled receptor 176 | 5.09 | 7.41E-03 |
| 12116 | *Bhmt* | betaine-homocysteine methyltransferase | 3.91 | 7.93E-03 |
| 230959 | *Ajap1* | adherens junction associated protein 1 | 3.62 | 9.63E-04 |
| 14573 | *Gdnf* | glial cell line derived neurotrophic factor | 3.43 | 9.63E-04 |
| 18113 | *Nnmt* | nicotinamide N-methyltransferase | 3.23 | 1.60E-02 |
| 229214 | *Qrfpr* | pyroglutamylated RFamide peptide receptor | 3.19 | 9.63E-04 |
| 237523 | *Ptprq* | protein tyrosine phosphatase, receptor type, Q | 3.16 | 9.63E-04 |
| 216613 | *Ccdc85a* | coiled-coil domain containing 85A | 3.04 | 5.09E-03 |
| 13924 | *Ptprv* | protein tyrosine phosphatase, receptor type, V | 3.02 | 9.63E-04 |
| 11656 | *Alas2* | aminolevulinic acid synthase 2, erythroid | 2.97 | 9.63E-04 |
| 102278 | *Cpne7* | copine VII | 2.94 | 9.63E-04 |
| 15129 | *Hbb-b1* | hemoglobin, beta adult major chain | 2.92 | 9.63E-04 |
| 12722 | *Clca1* | chloride channel calcium activated 1 | 2.91 | 9.63E-04 |
| 66166 | *S100a14* | S100 calcium binding protein A14 | 2.81 | 9.63E-04 |
| 110257 | *Hba-a2* | hemoglobin alpha, adult chain 2 | 2.66 | 9.63E-04 |
| 21957 | *Tnnt3* | troponin T3, skeletal, fast | 2.66 | 3.18E-02 |
| 68655 | *Fndc1* | fibronectin type III domain containing 1 | 2.45 | 2.54E-03 |
| 15122 | *Hba-a1* | hemoglobin alpha, adult chain 1 | 2.44 | 9.63E-04 |
| 260296 | *Trim61* | tripartite motif-containing 61 | 2.44 | 1.64E-02 |
| 80797 | *Clca2* | chloride channel calcium activated 2 | 2.41 | 3.66E-02 |

log2 FC: log_2_(fold-change), FDR: false-discovery rate

**Supplementary Table 5. Top 20 DEGs down-regulated by diabetes but not affected by pioglitazone treatment in glomeruli (Glom *db/db* only in *db/+* vs. *db/db* and *db/db* vs. *db/db* PIO).**

| GeneID | Symbol | Description | log2 FC | FDR |
| --- | --- | --- | --- | --- |
| 28248 | *Slco1a1* | solute carrier organic anion transporter family, member 1a1 | -3.31 | 9.63E-04 |
| 72301 | *1810041L15Rik* | RIKEN cDNA 1810041L15 gene | -3.01 | 9.63E-04 |
| 332942 | *Gm853* | predicted gene 853 | -2.98 | 2.16E-02 |
| 11846 | *Arg1* | arginase, liver | -2.92 | 1.45E-02 |
| 320840 | *Negr1* | neuronal growth regulator 1 | -2.81 | 9.63E-04 |
| 270893 | *Tmem132e* | transmembrane protein 132E | -2.54 | 9.63E-04 |
| 216166 | *Plk5* | polo-like kinase 5 | -2.46 | 2.54E-03 |
| 117167 | *Steap4* | STEAP family member 4 | -2.44 | 9.63E-04 |
| 230459 | *Cyp2j13* | cytochrome P450, family 2, subfamily j, polypeptide 13 | -2.03 | 9.63E-04 |
| 235402 | *Lingo1* | leucine rich repeat and Ig domain containing 1 | -1.97 | 9.63E-04 |
| 277468 | *Slc39a12* | solute carrier family 39 (zinc transporter), member 12 | -1.95 | 1.31E-02 |
| 208777 | *Sned1* | sushi, nidogen and EGF-like domains 1 | -1.93 | 9.63E-04 |
| 13409 | *Tmc1* | transmembrane channel-like gene family 1 | -1.91 | 1.17E-02 |
| 14584 | *Gfpt2* | glutamine fructose-6-phosphate transaminase 2 | -1.73 | 5.67E-03 |
| 20558 | *Slfn4* | schlafen 4 | -1.69 | 3.56E-02 |
| 15507 | *Hspb1* | heat shock protein 1 | -1.68 | 9.63E-04 |
| 12007 | *Azgp1* | alpha-2-glycoprotein 1, zinc | -1.64 | 2.99E-02 |
| 17858 | *Mx2* | myxovirus (influenza virus) resistance 2 | -1.62 | 3.87E-03 |
| 380930 | *9330188P03Rik* | RIKEN cDNA 9330188P03 gene | -1.62 | 9.63E-04 |
| 18030 | *Nfil3* | nuclear factor, interleukin 3, regulated | -1.62 | 6.26E-03 |

log2 FC: log_2_(fold-change), FDR: false-discovery rate

**Supplementary Table 6. Top 20 DEGs up-regulated by diabetes and regulated by pioglitazone treatment in sciatic nerve (*db/+* vs. *db/db* and *db/db* vs. *db/db* PIO).**

| GeneID | Symbol | Description | log2 FC | |
| --- | --- | --- | --- | --- |
|  |  |  | *db/+* vs. *db/db* | *db/db* vs. *db/db* PIO |
| 12478 | *Cd19* | CD19 antigen | 6.16 | -3.57 |
| 12482 | *Ms4a1* | membrane-spanning 4-domains, subfamily A, member 1 | 5.53 | -4.78 |
| 272382 | *Spib* | Spi-B transcription factor (Spi-1/PU.1 related) | 4.99 | -4.07 |
| 17879 | *Myh1* | myosin, heavy polypeptide 1, skeletal muscle, adult | 4.95 | 1.68 |
| 22780 | *Ikzf3* | IKAROS family zinc finger 3 | 4.94 | -3.40 |
| 381113 | *Cdkl4* | cyclin-dependent kinase-like 4 | 4.89 | -2.32 |
| 242341 | *Atp6v0d2* | ATPase, H+ transporting, lysosomal V0 subunit D2 | 4.83 | 1.29 |
| 24108 | *Ubd* | ubiquitin D | 4.61 | 3.09 |
| 12902 | *Cr2* | complement receptor 2 | 4.48 | -3.58 |
| 11501 | *Adam8* | a disintegrin and metallopeptidase domain 8 | 4.46 | 1.23 |
| 20201 | *S100a8* | S100 calcium binding protein A8 (calgranulin A) | 4.38 | 1.80 |
| 72049 | *Tnfrsf13c* | tumor necrosis factor receptor superfamily, member 13c | 4.35 | -4.24 |
| 50778 | *Rgs1* | regulator of G-protein signaling 1 | 4.27 | 0.85 |
| 63993 | *Slc5a7* | solute carrier family 5 (choline transporter), member 7 | 4.20 | 0.68 |
| 12775 | *Ccr7* | chemokine (C-C motif) receptor 7 | 4.09 | -2.68 |
| 12518 | *Cd79a* | CD79A antigen (immunoglobulin-associated alpha) | 4.09 | -3.11 |
| 17068 | *Ly6d* | lymphocyte antigen 6 complex, locus D | 4.06 | -3.91 |
| 22227 | *Ucp1* | uncoupling protein 1 (mitochondrial, proton carrier) | 3.83 | 6.24 |
| 12502 | *Cd3g* | CD3 antigen, gamma polypeptide | 3.82 | -3.68 |
| 60361 | *Ms4a4b* | membrane-spanning 4-domains, subfamily A, member 4B | 3.81 | -3.14 |

log2 FC: log_2_(fold-change)

**Supplementary Table 7.** **Top 20 DEGs down-regulated by diabetes and regulated by pioglitazone treatment in sciatic nerve (*db/+* vs. *db/db* and *db/db* vs. *db/db* PIO).**

| GeneID | Symbol | Description | log2 FC | |
| --- | --- | --- | --- | --- |
|  |  |  | *db/+* vs. *db/db* | *db/db* vs. *db/db* PIO |
| 100039008 | *Mup10* | major urinary protein 10 | -4.67 | 2.46 |
| 109828 | *C7* | complement component 7 | -4.58 | 2.58 |
| 20319 | *Sfrp2* | secreted frizzled-related protein 2 | -3.08 | 2.22 |
| 17395 | *Mmp9* | matrix metallopeptidase 9 | -2.96 | 1.06 |
| 330953 | *Hcn4* | hyperpolarization-activated, cyclic nucleotide-gated K+ 4 | -2.83 | 2.89 |
| 13106 | *Cyp2e1* | cytochrome P450, family 2, subfamily e, polypeptide 1 | -2.83 | 3.65 |
| 18383 | *Tnfrsf11b* | tumor necrosis factor receptor superfamily, member 11b (osteoprotegerin) | -2.63 | 1.58 |
| 108151 | *Sema3d* | sema domain, immunoglobulin domain (Ig), short basic domain, secreted, (semaphorin) 3D | -2.59 | 1.76 |
| 20423 | *Shh* | sonic hedgehog | -2.58 | -1.63 |
| 11537 | *Cfd* | complement factor D (adipsin) | -2.53 | -2.75 |
| 22414 | *Wnt2b* | wingless related MMTV integration site 2b | -2.41 | 2.01 |
| 50786 | *Hs6st2* | heparan sulfate 6-O-sulfotransferase 2 | -2.38 | 1.19 |
| 12845 | *Comp* | cartilage oligomeric matrix protein | -2.38 | 1.73 |
| 16979 | *Lrrn1* | leucine rich repeat protein 1, neuronal | -2.27 | 2.05 |
| 14264 | *Fmod* | fibromodulin | -2.22 | 1.52 |
| 12873 | *Cpa3* | carboxypeptidase A3, mast cell | -2.22 | 0.97 |
| 75677 | *Cldn22* | claudin 22 | -2.16 | 2.11 |
| 17227 | *Mcpt4* | mast cell protease 4 | -2.12 | 0.98 |
| 211652 | *Wwc1* | WW, C2 and coiled-coil domain containing 1 | -2.08 | 1.35 |
| 20379 | *Sfrp4* | secreted frizzled-related protein 4 | -2.06 | 1.62 |

log2 FC: log_2_(fold-change)

**Supplementary Table 8.** **Top 20 DEGs up-regulated by diabetes and regulated by pioglitazone treatment in glomeruli (*db/+* vs. *db/db* and *db/db* vs. *db/db* PIO).**

| GeneID | Symbol | Description | log2 FC | |
| --- | --- | --- | --- | --- |
|  |  |  | *db/+* vs. *db/db* | *db/db* vs. *db/db* PIO |
| 23892 | *Grem1* | gremlin 1 | 6.49 | -6.78 |
| 194352 | *Trpv5* | transient receptor potential cation channel, subfamily V, member 5 | 4.84 | -2.98 |
| 100689 | *Spon2* | spondin 2, extracellular matrix protein | 4.82 | -2.92 |
| 18781 | *Pla2g2c* | phospholipase A2, group IIC | 4.29 | -2.00 |
| 225642 | *Grp* | gastrin releasing peptide | 4.06 | -1.73 |
| 80733 | *Car15* | carbonic anhydrase 15 | 3.90 | -4.30 |
| 20568 | *Slpi* | secretory leukocyte peptidase inhibitor | 3.86 | -2.54 |
| 16669 | *Krt19* | keratin 19 | 3.85 | -4.91 |
| 433004 | *B830017H08Rik* | RIKEN cDNA B830017H08 gene | 3.75 | -4.35 |
| 16622 | *Klk1b5* | kallikrein 1-related peptidase b5 | 3.73 | -4.00 |
| 16509 | *Kcne1* | potassium voltage-gated channel, Isk-related subfamily, member 1 | 3.65 | -3.38 |
| 11944 | *Atp4a* | ATPase, H+/K+ exchanging, gastric, alpha polypeptide | 3.63 | -3.71 |
| 18039 | *Nefl* | neurofilament, light polypeptide | 3.59 | -5.27 |
| 11815 | *Apod* | apolipoprotein D | 3.57 | -1.87 |
| 23893 | *Grem2* | gremlin 2 homolog, cysteine knot superfamily (Xenopus laevis) | 3.49 | -6.91 |
| 330908 | *Opcml* | opioid binding protein/cell adhesion molecule-like | 3.44 | -0.81 |
| 20855 | *Stc1* | stanniocalcin 1 | 3.33 | -3.63 |
| 56739 | *Rec8* | REC8 homolog (yeast) | 3.30 | -3.65 |
| 13119 | *Cyp4a14* | cytochrome P450, family 4, subfamily a, polypeptide 14 | 3.30 | -0.90 |
| 20728 | *Spic* | Spi-C transcription factor (Spi-1/PU.1 related) | 3.28 | -1.74 |

log2 FC: log_2_(fold-change)

**Supplementary Table 9.** **Top 20 DEGs down-regulated by diabetes and regulated by pioglitazone treatment in glomeruli (*db/+* vs. *db/db* and *db/db* vs. *db/db* PIO).**

| GeneID | Symbol | Description | log2 FC | |
| --- | --- | --- | --- | --- |
|  |  |  | *db/+* vs. *db/db* | *db/db* vs. *db/db* PIO |
| 18143 | *Npas2* | neuronal PAS domain protein 2 | -3.57 | 1.44 |
| 263764 | *Creg2* | cellular repressor of E1A-stimulated genes 2 | -3.40 | 2.07 |
| 56808 | *Cacna2d2* | calcium channel, voltage-dependent, alpha 2/delta subunit 2 | -2.75 | 1.20 |
| 378435 | *Mafa* | v-maf musculoaponeurotic fibrosarcoma oncogene family, protein A (avian) | -2.46 | 1.37 |
| 11865 | *Arntl* | aryl hydrocarbon receptor nuclear translocator-like | -2.29 | 1.04 |
| 271970 | *Arsj* | arylsulfatase J | -2.10 | 0.86 |
| 268510 | *Mgat5b* | mannoside acetylglucosaminyltransferase 5, isoenzyme B | -2.08 | 0.95 |
| 72168 | *Aifm3* | apoptosis-inducing factor, mitochondrion-associated 3 | -1.95 | 1.08 |
| 244867 | *Arhgap20* | Rho GTPase activating protein 20 | -1.93 | 2.74 |
| 14563 | *Gdf5* | growth differentiation factor 5 | -1.73 | 1.16 |
| 16840 | *Lect1* | leukocyte cell derived chemotaxin 1 | -1.73 | 0.70 |
| 14181 | *Fgfbp1* | fibroblast growth factor binding protein 1 | -1.71 | 0.81 |
| 18124 | *Nr4a3* | nuclear receptor subfamily 4, group A, member 3 | -1.67 | 1.11 |
| 20681 | *Sox8* | SRY (sex determining region Y)-box 8 | -1.61 | 1.16 |
| 14611 | *Gja3* | gap junction protein, alpha 3 | -1.57 | 0.56 |
| 67775 | *Rtp4* | receptor transporter protein 4 | -1.54 | 0.95 |
| 16510 | *Kcnh1* | potassium voltage-gated channel, subfamily H (eag-related), member 1 | -1.53 | 1.05 |
| 19217 | *Ptger2* | prostaglandin E receptor 2 (subtype EP2) | -1.49 | 0.96 |
| 224997 | *Dlgap1* | discs, large (Drosophila) homolog-associated protein 1 | -1.48 | 1.29 |
| 320158 | *Zmat4* | zinc finger, matrin type 4 | -1.47 | 0.69 |

log2 FC: log_2_(fold-change)

**Supplementary Table 10. Top 20 shared genes between down-regulated by diabetes and reversed by pioglitazone treatment in glomeruli and only regulated by diabetes in SCN (down-regulated Glom Reversed vs. SCN *db/db* only) (sorted by fold-change of *db/db* vs. *db/db* PIO in glomeruli).**

| GeneID | Symbol | Description | Glomeruli log2 FC | | SCN log2 FC |
| --- | --- | --- | --- | --- | --- |
|  |  |  | *db/+* vs. *db/db* | *db/db* vs. *db/db* PIO | *db/+* vs. *db/db* |
| 54635 | *Pdgfc* | platelet-derived growth factor, C polypeptide | -0.88 | 1.31 | 1.30 |
| 55932 | *Gbp3* | guanylate binding protein 3 | -0.65 | 1.12 | -0.53 |
| 11865 | *Arntl* | aryl hydrocarbon receptor nuclear translocator-like | -2.29 | 1.04 | -0.53 |
| 14469 | *Gbp2* | guanylate binding protein 2 | -0.69 | 1.03 | -0.48 |
| 93737 | *Pard6g* | par-6 family cell polarity regulator gamma | -0.69 | 1.01 | 0.51 |
| 67775 | *Rtp4* | receptor transporter protein 4 | -1.54 | 0.95 | -0.90 |
| 243385 | *Gprin3* | GPRIN family member 3 | -1.13 | 0.90 | 1.56 |
| 26427 | *Creb3l1* | cAMP responsive element binding protein 3-like 1 | -1.12 | 0.80 | -0.86 |
| 16194 | *Il6ra* | interleukin 6 receptor, alpha | -0.48 | 0.80 | 0.67 |
| 327959 | *Xaf1* | XIAP associated factor 1 | -0.81 | 0.71 | -0.48 |
| 233271 | *Luzp2* | leucine zipper protein 2 | -1.19 | 0.68 | -0.53 |
| 235435 | *Lctl* | lactase-like | -1.08 | 0.66 | -0.51 |
| 73173 | *Pcdh18* | protocadherin 18 | -0.54 | 0.63 | -0.96 |
| 17965 | *Nbl1* | neuroblastoma, suppression of tumorigenicity 1 | -0.58 | 0.62 | -0.52 |
| 67198 | *Spats2l* | spermatogenesis associated, serine-rich 2-like | -0.87 | 0.59 | -1.26 |
| 74091 | *Npl* | N-acetylneuraminate pyruvate lyase | -1.43 | 0.59 | -0.90 |
| 14051 | *Eya4* | eyes absent 4 homolog (Drosophila) | -0.71 | 0.58 | 0.69 |
| 14561 | *Gdf11* | growth differentiation factor 11 | -0.86 | 0.56 | -0.76 |
| 100702 | *Gbp6* | guanylate binding protein 6 | -0.69 | 0.54 | -0.63 |
| 24055 | *Sh3bp2* | SH3-domain binding protein 2 | -0.65 | 0.53 | 0.59 |

log2 FC: log_2_(fold-change)

**Supplementary Table 11. Top 20 shared genes between up-regulated by diabetes and reversed by pioglitazone treatment in glomeruli and only regulated by diabetes in SCN (up-regulated Glom Reversed vs. SCN *db/db* only) (sorted by fold-change of *db/db* vs. *db/db* PIO in glomeruli).**

| GeneID | Symbol | Description | Glomeruli log2 FC | | SCN log2 FC |
| --- | --- | --- | --- | --- | --- |
|  |  |  | *db/+* vs. *db/db* | *db/db* vs. *db/db* PIO | *db/+* vs. *db/db* |
| 12035 | *Bcat1* | branched chain aminotransferase 1, cytosolic | 2.74 | -4.37 | 0.90 |
| 20750 | *Spp1* | secreted phosphoprotein 1 | 1.91 | -3.81 | -1.07 |
| 12223 | *Btc* | betacellulin, epidermal growth factor family member | 2.51 | -3.72 | 1.55 |
| 64292 | *Ptges* | prostaglandin E synthase | 2.53 | -3.58 | -0.55 |
| 75104 | *Mmd2* | monocyte to macrophage differentiation-associated 2 | 2.37 | -3.54 | -1.01 |
| 14411 | *Slc6a12* | solute carrier family 6 (neurotransmitter transporter, betaine/GABA), member 12 | 2.59 | -3.25 | -1.04 |
| 75590 | *Dusp9* | dual specificity phosphatase 9 | 1.90 | -3.24 | 3.53 |
| 11522 | *Adh1* | alcohol dehydrogenase 1 (class I) | 2.56 | -3.13 | 0.49 |
| 11936 | *Fxyd2* | FXYD domain-containing ion transport regulator 2 | 0.90 | -3.11 | 0.82 |
| 12842 | *Col1a1* | collagen, type I, alpha 1 | 1.33 | -3.08 | -0.77 |
| 56013 | *Srcin1* | SRC kinase signaling inhibitor 1 | 2.40 | -3.07 | -0.89 |
| 16763 | *Lad1* | ladinin | 2.42 | -3.04 | -1.17 |
| 57890 | *Il17re* | interleukin 17 receptor E | 2.17 | -2.99 | -1.26 |
| 66889 | *Rnf128* | ring finger protein 128 | 1.70 | -2.93 | 0.40 |
| 100689 | *Spon2* | spondin 2, extracellular matrix protein | 4.82 | -2.92 | -0.56 |
| 18260 | *Ocln* | occludin | 1.65 | -2.89 | -0.97 |
| 71897 | *Lypd6b* | LY6/PLAUR domain containing 6B | 2.16 | -2.84 | -1.30 |
| 56792 | *Stap1* | signal transducing adaptor family member 1 | 1.75 | -2.84 | 2.89 |
| 16525 | *Kcnk1* | potassium channel, subfamily K, member 1 | 1.66 | -2.69 | 0.42 |
| 320563 | *Islr2* | immunoglobulin superfamily containing leucine-rich repeat 2 | 1.83 | -1.57 | -1.36 |

log2 FC: log_2_(fold-change)

**Supplementary Table 12. Shared genes between down-regulated by diabetes and reversed by pioglitazone treatment in glomeruli and exacerbated by pioglitazone treatment in SCN (down-regulated Glom Reversed vs. SCN exacerbated) (sorted by fold change of *db/db* vs. *db/db* PIO in glomeruli).**

| GeneID | Symbol | Description | Glomeruli log2 FC | | SCN log2 FC | |
| --- | --- | --- | --- | --- | --- | --- |
|  |  |  | *db/+* vs. *db/db* | *db/db* vs. *db/db* PIO | *db/+* vs. *db/db* | *db/db* vs. *db/db* PIO |
| 381339 | *Tmem182* | transmembrane protein 182 | -1.04 | 1.20 | 2.14 | 0.73 |
| 19217 | *Ptger2* | prostaglandin E receptor 2 (subtype EP2) | -1.49 | 0.96 | 1.17 | 0.82 |
| 18162 | *Npr3* | natriuretic peptide receptor 3 | -0.75 | 0.81 | 1.94 | 0.62 |
| 16840 | *Lect1* | leukocyte cell derived chemotaxin 1 | -1.73 | 0.70 | -0.58 | -0.61 |
| 14457 | *Gas7* | growth arrest specific 7 | -0.71 | 0.60 | -0.39 | -0.28 |
| 19242 | *Ptn* | pleiotrophin | -0.96 | 0.52 | -0.46 | -0.67 |
| 330319 | *Wipf3* | WAS/WASL interacting protein family, member 3 | -0.55 | 0.51 | 0.91 | 0.90 |
| 16658 | *Mafb* | v-maf musculoaponeurotic fibrosarcoma oncogene family, protein B (avian) | -0.62 | 0.46 | 1.19 | 0.87 |
| 433375 | *Creg1* | cellular repressor of E1A-stimulated genes 1 | -0.49 | 0.39 | 0.31 | 0.41 |

log2 FC: log_2_(fold-change)

**Supplementary Table 13. Top 20 shared genes between up-regulated by diabetes and reversed by pioglitazone treatment in glomeruli and exacerbated by pioglitazone treatment in SCN (up-regulated Glom Reversed vs. SCN exacerbated) (sorted by fold change of *db/db* vs. *db/db* PIO in glomeruli).**

| GeneID | Symbol | Description | Glomeruli log2 FC | | SCN log2 FC | |
| --- | --- | --- | --- | --- | --- | --- |
|  |  |  | *db/+* vs. *db/db* | *db/db* vs. *db/db* PIO | *db/+* vs. *db/db* | *db/db* vs. *db/db* PIO |
| 242341 | *Atp6v0d2* | ATPase, H+ transporting, lysosomal V0 subunit D2 | 2.67 | -3.02 | 4.83 | 1.29 |
| 245945 | *Rbm47* | RNA binding motif protein 47 | 1.74 | -2.87 | 0.74 | 1.14 |
| 22239 | *Ugt8a* | UDP galactosyltransferase 8A | 1.52 | -2.85 | -0.48 | -0.40 |
| 104943 | *Fam110c* | family with sequence similarity 110, member C | 1.40 | -2.69 | 3.05 | 1.16 |
| 15446 | *Hpgd* | hydroxyprostaglandin dehydrogenase 15 (NAD) | 1.77 | -2.66 | 1.00 | 0.64 |
| 235135 | *Tmem45b* | transmembrane protein 45b | 2.09 | -2.66 | 0.78 | 0.36 |
| 12550 | *Cdh1* | cadherin 1 | 1.86 | -2.65 | -0.42 | -0.53 |
| 16728 | *L1cam* | L1 cell adhesion molecule | 2.03 | -2.42 | -0.31 | -0.34 |
| 16854 | *Lgals3* | lectin, galactose binding, soluble 3 | 2.15 | -2.41 | 2.12 | 0.60 |
| 17060 | *Blnk* | B cell linker | 2.03 | -2.40 | 2.40 | 0.52 |
| 83433 | *Trem2* | triggering receptor expressed on myeloid cells 2 | 2.72 | -2.39 | 3.05 | 1.05 |
| 14131 | *Fcgr3* | Fc receptor, IgG, low affinity III | 1.04 | -2.34 | 0.70 | 0.75 |
| 12759 | *Clu* | clusterin | 1.23 | -2.32 | -0.33 | -0.49 |
| 13723 | *Emb* | embigin | 1.61 | -2.14 | 1.33 | 1.07 |
| 74559 | *Elovl7* | ELOVL family member 7, elongation of long chain fatty acids (yeast) | 1.27 | -2.13 | -0.45 | -0.30 |
| 227960 | *Gca* | grancalcin | 1.90 | -2.12 | -0.34 | -0.38 |
| 20135 | *Rrm2* | ribonucleotide reductase M2 | 1.61 | -2.11 | 1.85 | 0.92 |
| 19229 | *Ptk2b* | PTK2 protein tyrosine kinase 2 beta | 1.16 | -2.11 | 1.38 | 0.63 |
| 16590 | *Kit* | kit oncogene | 1.93 | -2.06 | 0.73 | 0.41 |
| 11630 | *Aim1* | absent in melanoma 1 | 1.88 | -2.04 | 0.82 | 0.49 |

log2 FC: log_2_(fold-change)

**Supplementary Table 14. Top 20 shared genes between down-regulated by diabetes and reversed by pioglitazone treatment in glomeruli and reversed by pioglitazone treatment in SCN (down-regulated Glom Reversed vs. SCN Reversed) (sorted by fold change of *db/db* vs. *db/db* PIO in glomeruli).**

| GeneID | Symbol | Description | Glomeruli log2 FC | | SCN log2 FC | |
| --- | --- | --- | --- | --- | --- | --- |
|  |  |  | *db/+* vs. *db/db* | *db/db* vs. *db/db* PIO | *db/+* vs. *db/db* | *db/db* vs. *db/db* PIO |
| 244867 | *Arhgap20* | Rho GTPase activating protein 20 | -1.93 | 2.74 | -0.79 | 0.67 |
| 18143 | *Npas2* | neuronal PAS domain protein 2 | -3.57 | 1.44 | -1.15 | 1.23 |
| 12552 | *Cdh11* | cadherin 11 | -1.25 | 1.20 | -0.90 | 0.88 |
| 20319 | *Sfrp2* | secreted frizzled-related protein 2 | -1.16 | 0.98 | -3.08 | 2.22 |
| 17533 | *Mrc1* | mannose receptor, C type 1 | -0.77 | 0.97 | -0.40 | 0.82 |
| 26563 | *Ror1* | receptor tyrosine kinase-like orphan receptor 1 | -0.67 | 0.89 | -1.14 | 0.80 |
| 258571 | *Olfr1033* | olfactory receptor 1033 | -0.86 | 0.78 | -0.81 | 0.82 |
| 330096 | *Shisa3* | shisa homolog 3 (Xenopus laevis) | -0.58 | 0.67 | -1.33 | 1.08 |
| 70727 | *Rasgef1a* | RasGEF domain family, member 1A | -1.21 | 0.67 | 0.31 | -0.43 |
| 268860 | *Abat* | 4-aminobutyrate aminotransferase | -0.48 | 0.65 | -0.63 | 0.57 |
| 109700 | *Itga1* | integrin alpha 1 | -0.48 | 0.63 | -0.64 | 0.54 |
| 68813 | *Dock5* | dedicator of cytokinesis 5 | -0.67 | 0.55 | -0.59 | 0.40 |
| 399558 | *Flrt2* | fibronectin leucine rich transmembrane protein 2 | -0.90 | 0.55 | -2.05 | 1.54 |
| 104086 | *Cyp27a1* | cytochrome P450, family 27, subfamily a, polypeptide 1 | -0.70 | 0.54 | -0.46 | 0.50 |
| 22329 | *Vcam1* | vascular cell adhesion molecule 1 | -0.71 | 0.48 | -0.67 | 0.90 |
| 240725 | *Sulf1* | sulfatase 1 | -1.09 | 0.45 | -0.97 | 0.34 |
| 13482 | *Dpp4* | dipeptidylpeptidase 4 | -0.67 | 0.45 | -1.37 | 0.50 |
| 207521 | *Dtx4* | deltex 4 homolog (Drosophila) | -0.80 | 0.43 | -0.57 | 0.54 |
| 207259 | *Zbtb7c* | zinc finger and BTB domain containing 7C | -0.46 | 0.43 | -0.75 | 0.65 |
| 234353 | *Psd3* | pleckstrin and Sec7 domain containing 3 | -0.82 | 0.42 | -0.48 | 0.44 |

log2 FC: log_2_(fold-change)

**Supplementary Table 15. Top 20 shared genes between up-regulated by diabetes and reversed by pioglitazone treatment in glomeruli and reversed by pioglitazone treatment in SCN (up-regulated Glom Reversed vs. SCN Reversed) (sorted by fold change of *db/db* vs. *db/db* PIO in glomeruli).**

| GeneID | Symbol | Description | Glomeruli log2 FC | | SCN log2 FC | |
| --- | --- | --- | --- | --- | --- | --- |
|  |  |  | *db/+* vs. *db/db* | *db/db* vs. *db/db* PIO | *db/+* vs. *db/db* | *db/db* vs. *db/db* PIO |
| 110075 | *Bmp3* | bone morphogenetic protein 3 | 3.28 | -3.99 | -0.86 | 0.93 |
| 385643 | *Kng2* | kininogen 2 | 0.95 | -3.15 | -1.05 | 1.62 |
| 319767 | *Atp10b* | ATPase, class V, type 10B | 1.95 | -3.14 | 0.64 | -0.55 |
| 226049 | *Dmrt2* | doublesex and mab-3 related transcription factor 2 | 2.44 | -2.88 | 1.79 | -0.71 |
| 242721 | *Klhdc7a* | kelch domain containing 7A | 1.61 | -2.79 | -0.54 | 0.56 |
| 12902 | *Cr2* | complement receptor 2 | 2.66 | -2.77 | 4.48 | -3.58 |
| 13844 | *Ephb2* | Eph receptor B2 | 1.34 | -2.71 | -0.79 | 0.58 |
| 13179 | *Dcn* | decorin | 1.40 | -2.55 | -1.26 | 0.54 |
| 211652 | *Wwc1* | WW, C2 and coiled-coil domain containing 1 | 1.91 | -2.53 | -2.08 | 1.35 |
| 16782 | *Lamc2* | laminin, gamma 2 | 2.27 | -2.50 | -1.11 | 0.84 |
| 20348 | *Sema3c* | sema domain, immunoglobulin domain (Ig), short basic domain, secreted, (semaphorin) 3C | 1.55 | -2.47 | -0.79 | 0.65 |
| 380711 | *Rap1gap2* | RAP1 GTPase activating protein 2 | 1.24 | -2.43 | 1.14 | -0.50 |
| 12833 | *Col6a1* | collagen, type VI, alpha 1 | 1.54 | -2.35 | -0.62 | 0.94 |
| 224833 | *AI661453* | expressed sequence AI661453 | 1.84 | -2.34 | -1.33 | 0.77 |
| 19221 | *Ptgfrn* | prostaglandin F2 receptor negative regulator | 1.26 | -2.13 | -0.70 | 0.66 |
| 12825 | *Col3a1* | collagen, type III, alpha 1 | 0.96 | -2.09 | -1.26 | 0.78 |
| 20354 | *Sema4d* | sema domain, immunoglobulin domain (Ig), transmembrane domain (TM) and short cytoplasmic domain, (semaphorin) 4D | 0.97 | -2.08 | 1.08 | -0.40 |
| 15360 | *Hmgcs2* | 3-hydroxy-3-methylglutaryl-Coenzyme A synthase 2 | 0.70 | -1.76 | 0.37 | -0.86 |
| 19017 | *Ppargc1a* | peroxisome proliferative activated receptor, gamma, coactivator 1 alpha | 1.00 | -1.73 | -0.55 | 0.49 |
| 320563 | *Islr2* | immunoglobulin superfamily containing leucine-rich repeat 2 | 1.83 | -1.57 | -1.36 | 1.01 |

log2 FC: log_2_(fold-change)**Supplementary Table 16. Mouse primer sequences and gene expression validation of RNA-Seq data using RT-qPCR.**

| **Gene Symbol** | **Gene ID#** | **Primer Sequence** | | **RT-qPCR FC vs. Glom_Cont** | | **RT-qPCR FC vs. SCN_Cont** | |
| --- | --- | --- | --- | --- | --- | --- | --- |
|  | | | | **Glom_*db/db*** | **Glom_*db/db*-Pio** | **SCN_*db/db*** | **SCN_*db/db*-Pio** |
| *Ywhaz* | 22631 | Forward | AAGACAGCACGACGCTAATAATGC | N/A | N/A | N/A | N/A |
|  |  | Reverse | TTGGAAGGCCGGTTAATTTTC |  |  |  |  |
| *Acaa2* | 52538 | Forward | ACGTGAACGAAGCTTTTGCC | 1.727*** | 0.9651^###^ | N/D | N/D |
|  |  | Reverse | TTCCACCTCGACGCCTTAAC |  |  |  |  |
| *Echs1* | 93747 | Forward | CAACCAAGCACTGGAGACCT | 1.487** | 0.9243^##^ | N/D | N/D |
|  |  | Reverse | AACCCCCACCAAGAGCATAA |  |  |  |  |
| *Sdhb* | 67680 | Forward | TGCAGTTTCAGGCCTGTCGAG | 1.225* | 0.9169^###^ | 0.8008 | 1.443^###^ |
|  |  | Reverse | ACCATAGGTCCGCACTTATTCAG |  |  |  |  |
| *Cox4i1* | 12857 | Forward | GCCTTGGACGGCGGAAT | 1.749*** | 0.9802^###^ | 0.7105 | 1.825^###^ |
|  |  | Reverse | AACACTCCCATGTGCTCGAA |  |  |  |  |
| *Prdx5* | 54683 | Forward | CCGATCAAGGTGGGAGATGC | 2.373*** | 1.177^###^ | N/D | N/D |
|  |  | Reverse | TTAACGCTCAGACAGGCCAC |  |  |  |  |

Table indicates the fold-change (FC) of genes relative to *db/+* glomeruli (Glom_Cont) or *db/+* sciatic nerve (SCN_Cont), normalized to the geometric mean of endogenous control, tyrosine 3-monooxygenase/tryptophan 5-monooxygenase activation protein (*Ywhaz*). N/A, not applicable; ND, not determined. * p<0.05 vs. *db/+****,*** ** p<0.01 vs. *db/+,* *** p<0.001 vs. *db/+*; ^##^ p<0.01, ^###^ p<0.001 vs. *db/db*

**Supplementary Figure 1. Metabolic phenotyping.** (A) Body weight, (B) fasting blood glucose, (C) glycated hemoglobin, (D) fasting plasma insulin, (E) total fasting plasma cholesterol (F) total fasting plasma triglycerides were measured at 16 wk of age in all mice. *, p<0.05, **, p<0.01, ***, p<0.001 vs. *db/+*; ### p<0.001 vs. *db/db.*

**
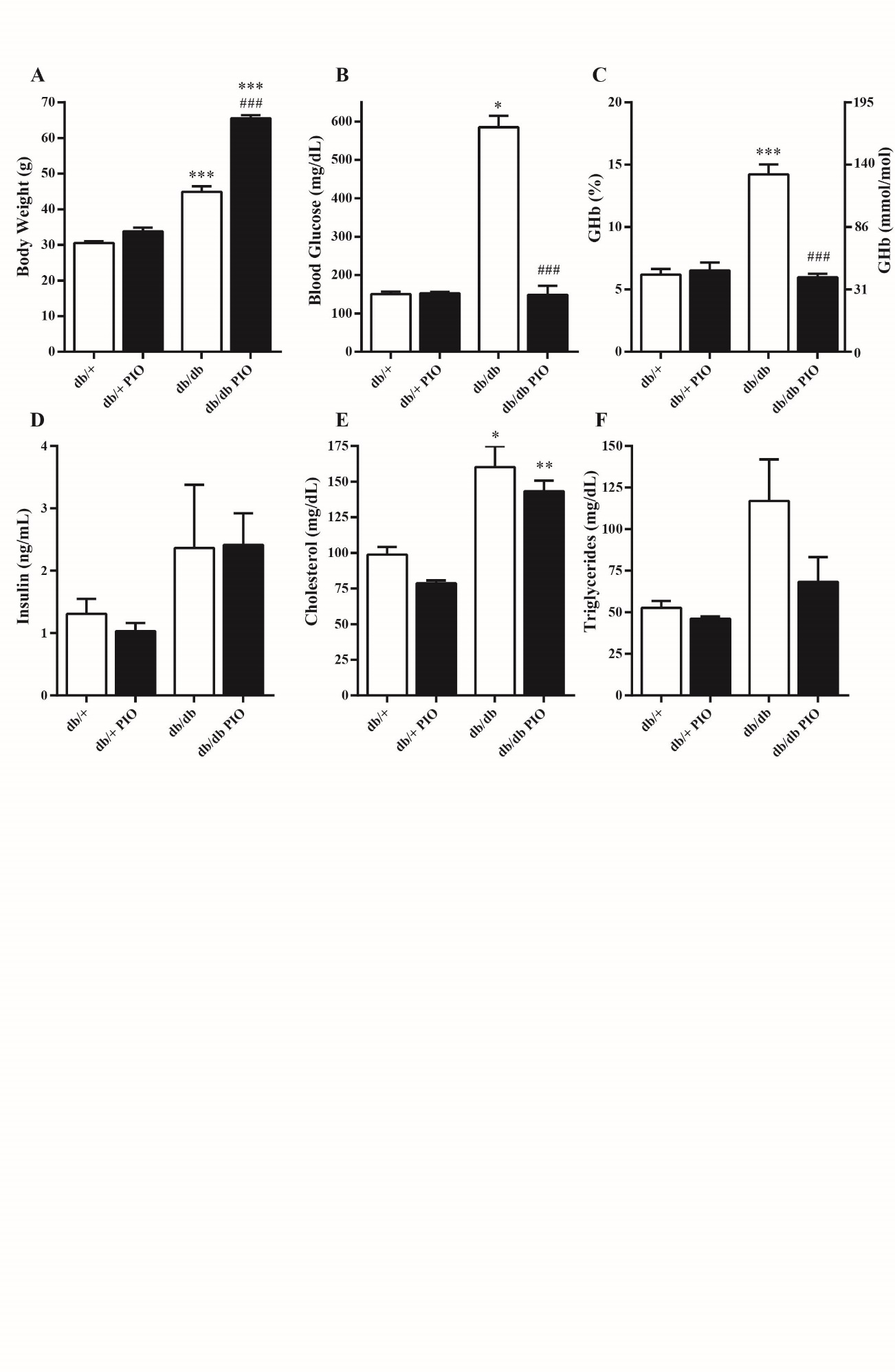
**

**Supplementary Figure 2. Neuropathy phenotyping.** (A) Hind paw withdrawal latency, (B) sciatic motor nerve conduction velocity (NCV), and (C) sural sensory NCV were measured at 16 wk of age in all mice. **, p<0.01, ***, p<0.001 vs. *db/+*; ##, p<0.01 vs. *db/db*.

**
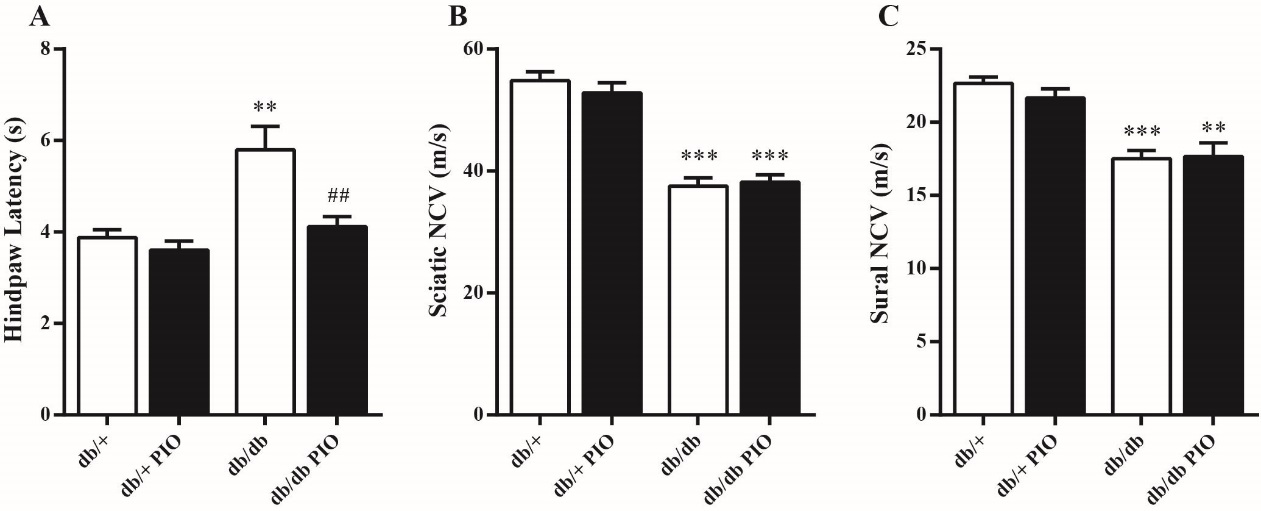
**

**Supplementary Figure 3. Nephropathy phenotyping.** (A) Glomerular Periodic-Acid Schiff (PAS)-positive area, (B) glomerular area, (C) mesangial index, and (D) albumin/creatinine ratio were measured at 16 wk of age in all mice. ***, p<0.001 vs. *db/+*; ##, p<0.01, ###, p<0.001 vs. *db/db*.

**
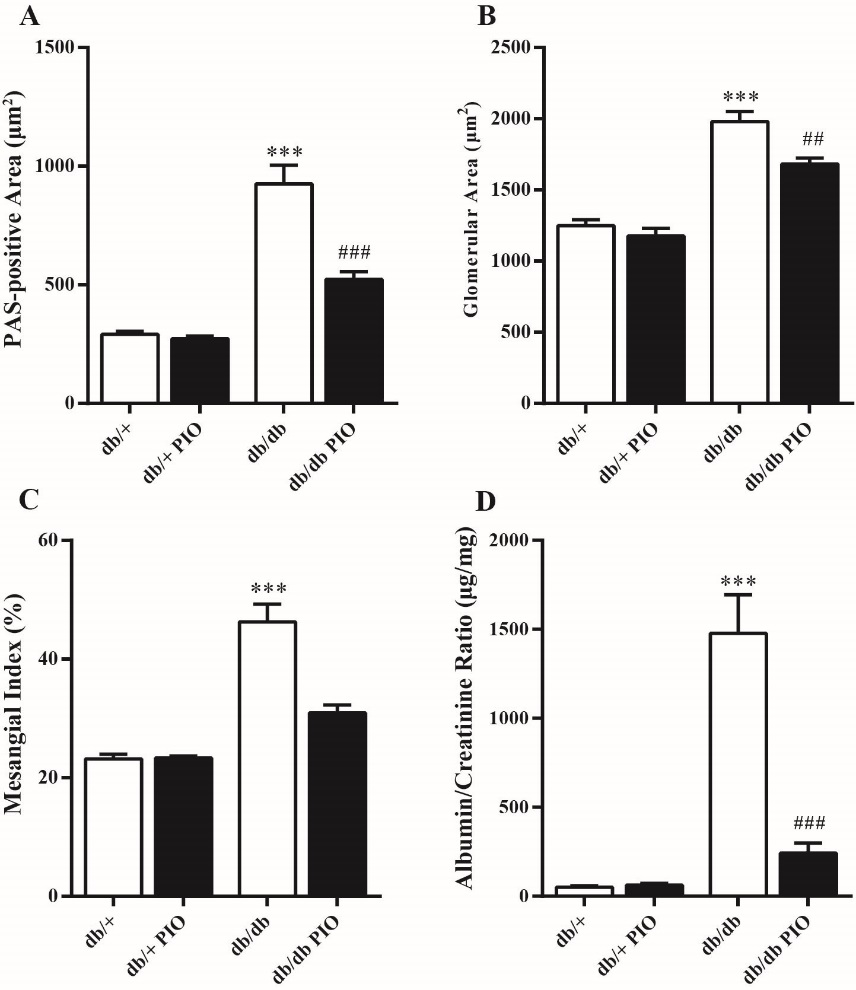
**

**Supplementary Figure 4. Effects of pioglitazone on kidney function.** Following 11 weeks of pioglitazone treatment, (A) 24-hour urinary albumin excretion, (B) urinary creatinine excretion, and (C) urinary volume were assessed. * p<0.05, *** p<0.001 vs. *db/+*; ### p<0.001 vs. *db/db*. In all panels, n = 7.


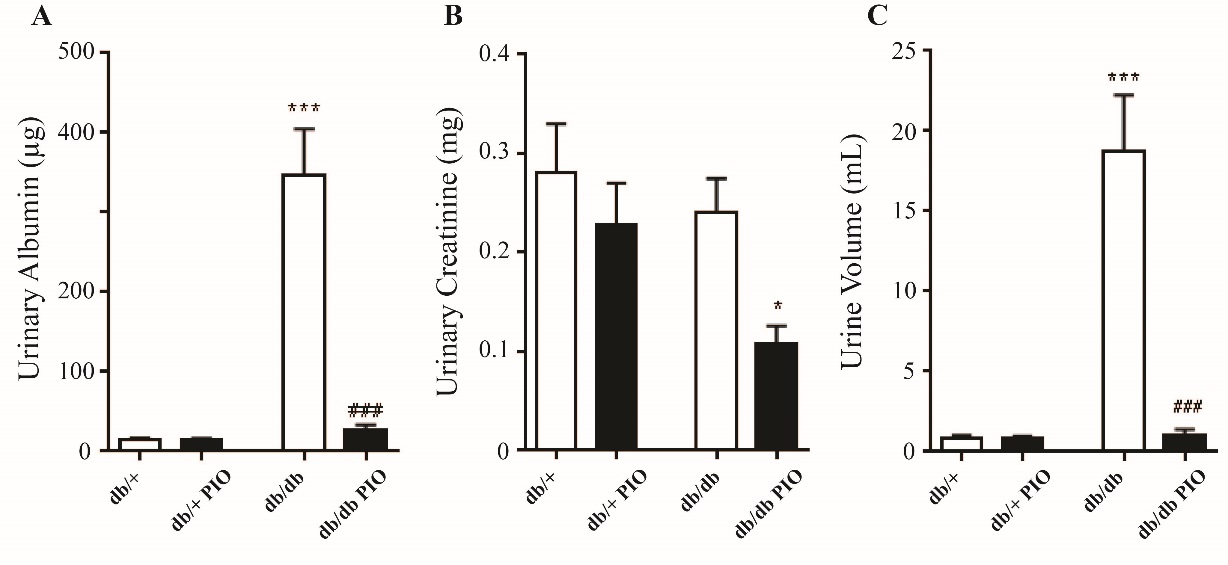


**Supplementary Figure 5. Correlation analysis.** A. Pearson correlation matrix of glycemia, small nerve fiber dysfunction (hind paw thermal withdrawal latency), and DN (mesangial index; albumin/creatinine ratio, ACR). Numbers represent correlation coefficients. B. Correlation plot of small fiber dysfunction and glomerular mesangial index (DN) (r = 0.8; P < 0.001).

**
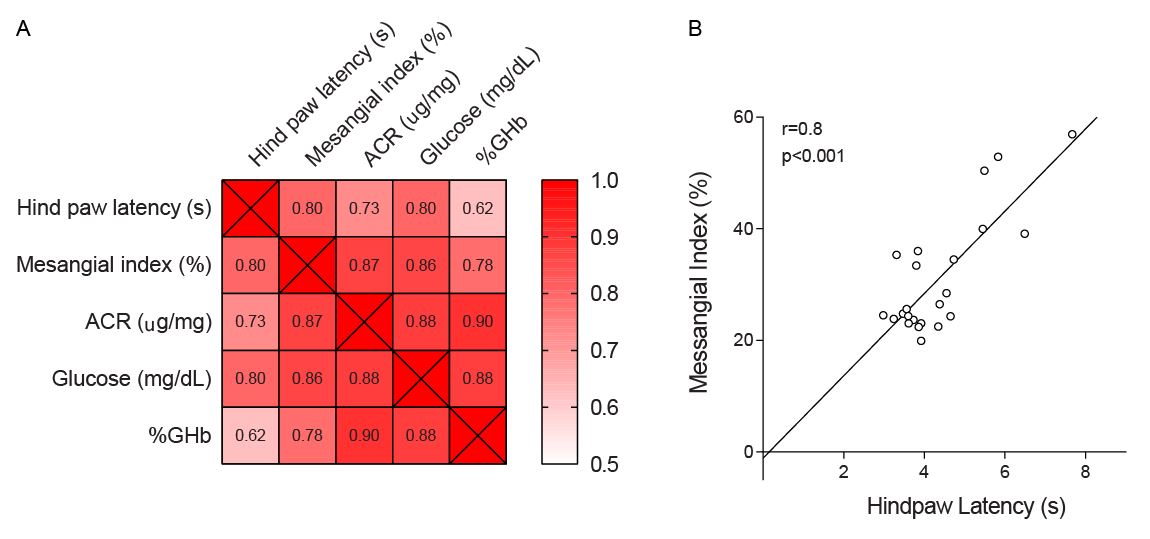
**

**Supplementary Figure 6. Log_2_(x+1) FKPM of PPAR isoforms across tissues.** The average FPKM values of all annotated transcripts of *Ppara*, *Ppard* and *Pparg* in our dataset. *Ppara* transcripts were expressed in kidney cortex, and *Ppard* and *Pparg* were expressed in glomeruli and SCN. In DRG, only *Ppard* was expressed, at low levels.


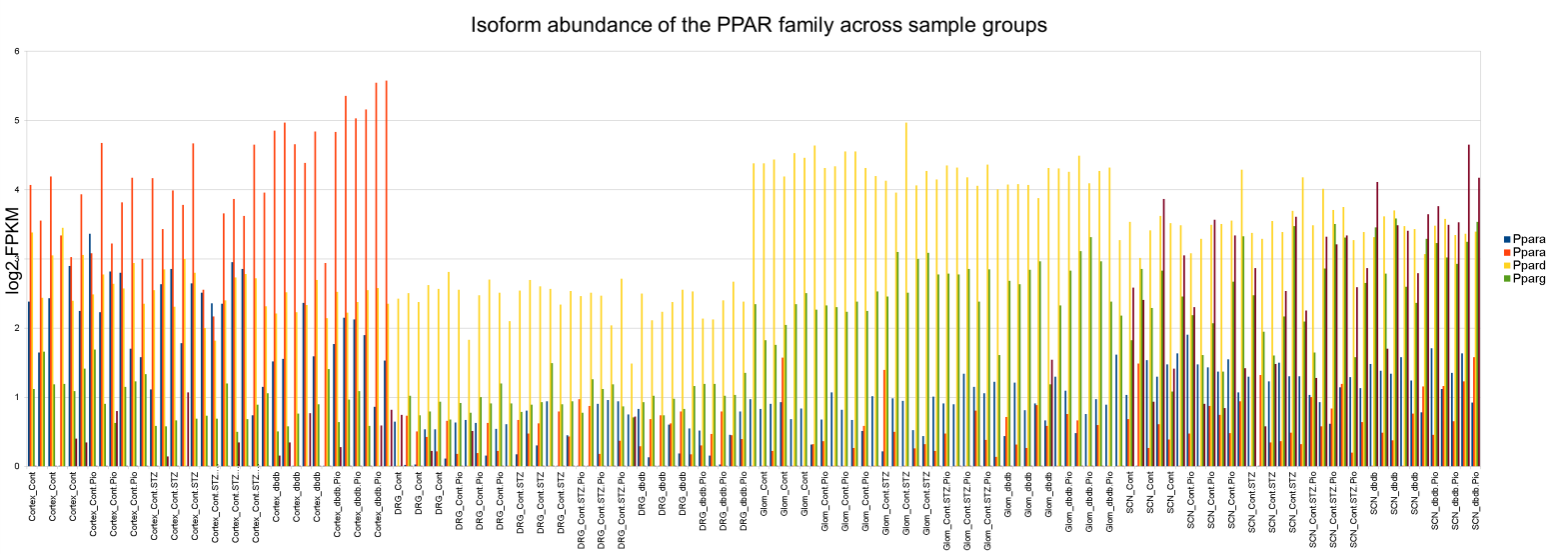


**Supplementary Figure 7. Functional Enrichment Analysis for all 49 modules from SOM analysis.** In order to investigate the biological functions enriched for all 49 modules, a functional enrichment analysis using DAVID (http://david.abcc.ncifcrf.gov/) was performed. The top 10 most significant functional terms for all modules were represented in a heat-map with a -log_10_(Benjamini-Hochberg corrected P-value) color-gradient.


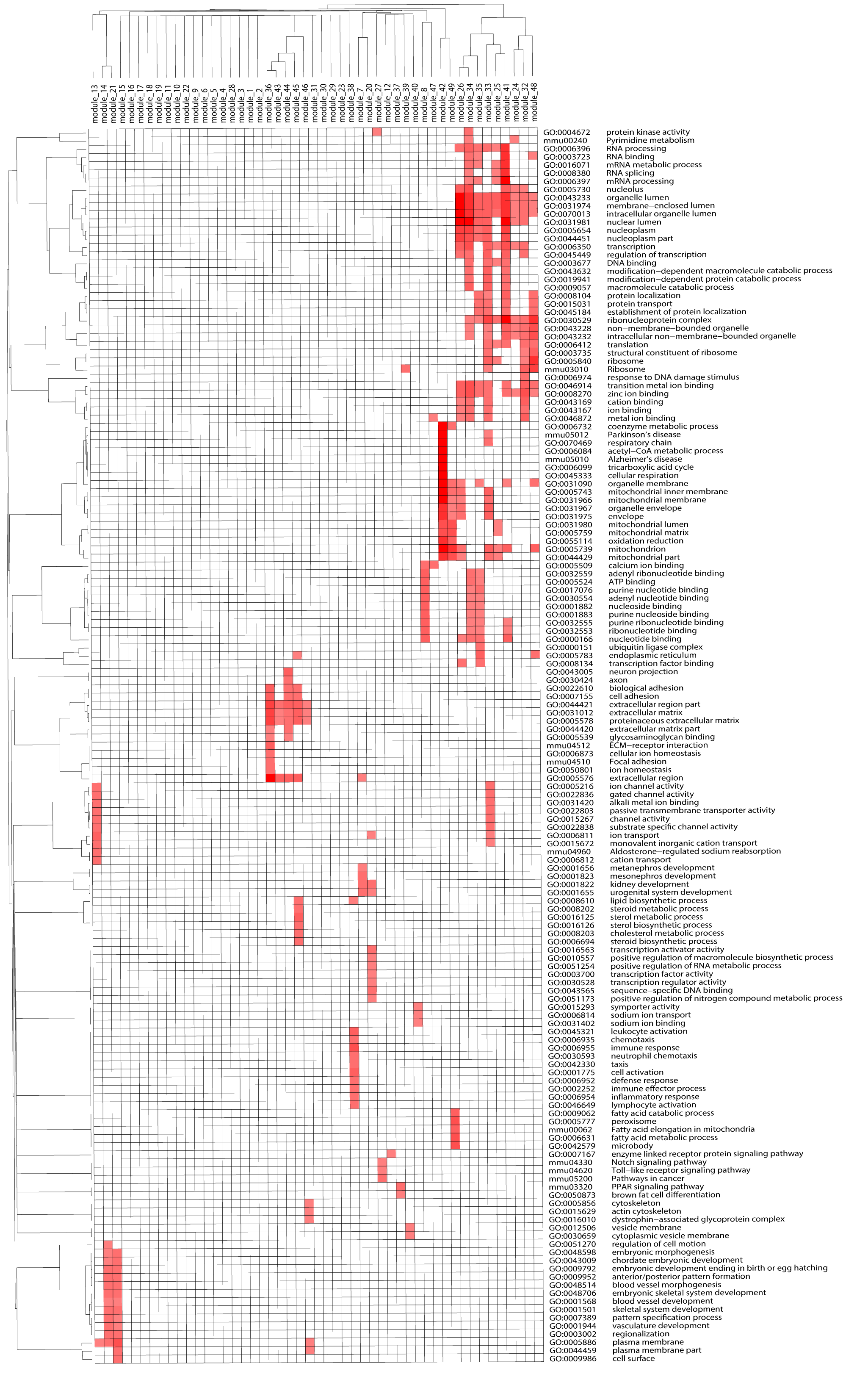


**Supplementary Figure 8. Comparisons of DEG sets from microarray and RNA-Seq analyses.** In order to investigate the difference between transcriptomic study results from our previous study and the current study, the DEG sets in nerve tissue were compared. (A) Overlapping DEGs between microarray and RNA-Seq analysis. (B) The fold-changes of common DEGs affected by diabetes and pioglitazone treatment from both platforms were examined. (C) Functional enrichment analysis using DAVID (http://david.abcc.ncifcrf.gov/) was performed on SCN. The top 20 most significant functional terms for all modules were represented in a heat-map with a -log_10_ (Benjamini-Hochberg corrected P-value) color-gradient.

_
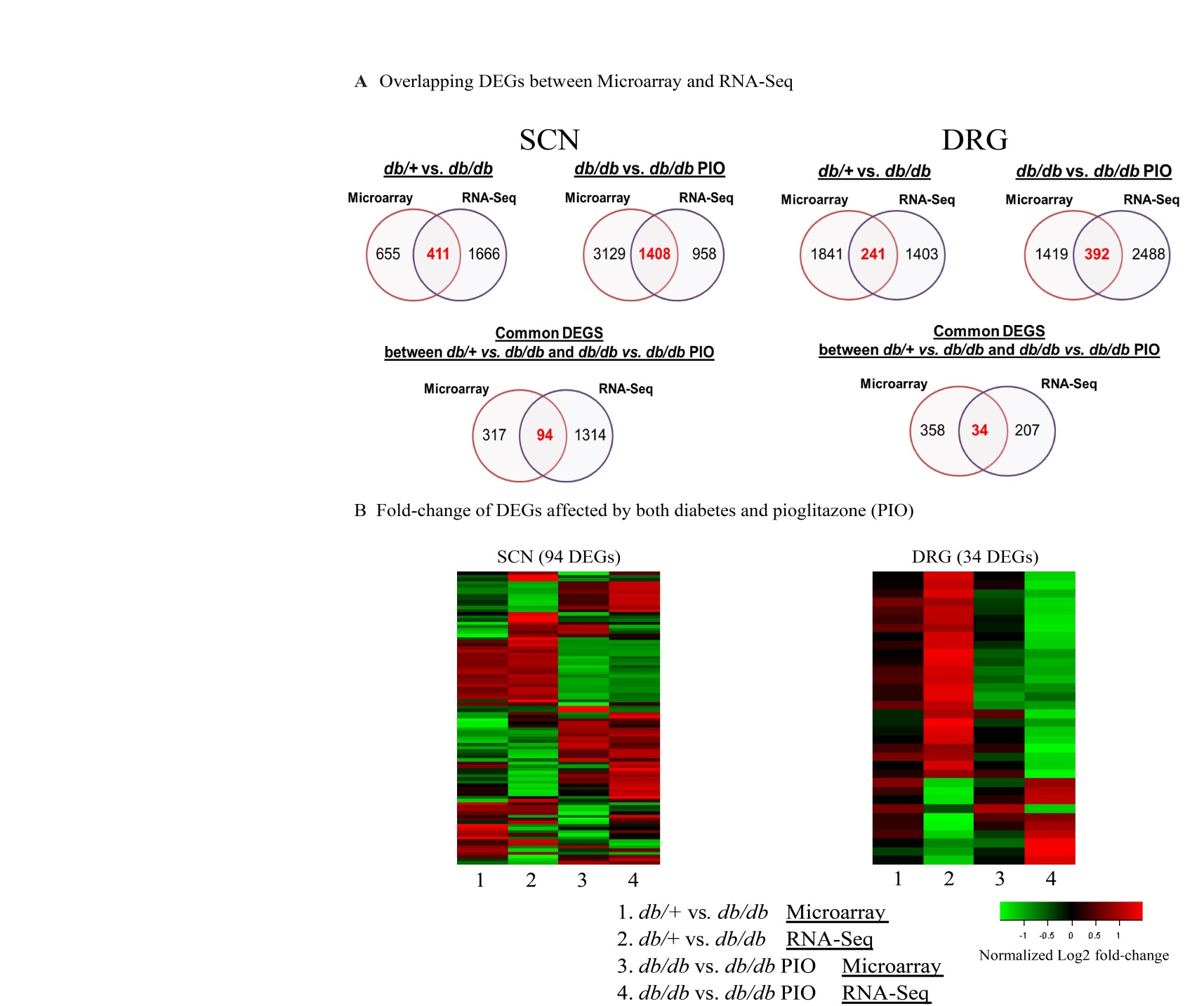
_

_
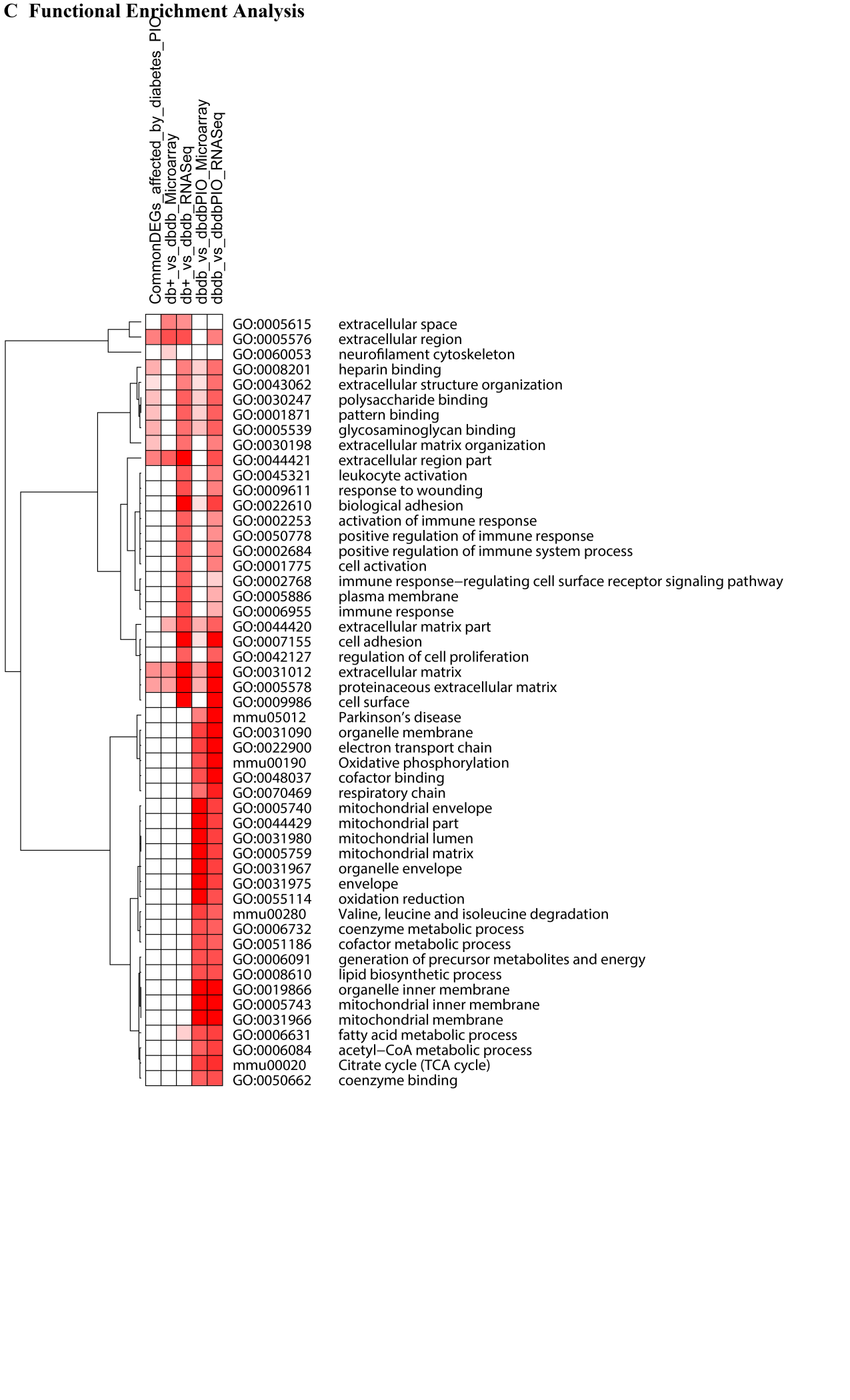
_
